# Conformational Changes of Anoplin, W-MreB_1–9_, and (KFF)_3_K Peptides near the Membranes

**DOI:** 10.3390/ijms21249672

**Published:** 2020-12-18

**Authors:** Monika Wojciechowska, Joanna Miszkiewicz, Joanna Trylska

**Affiliations:** 1 Centre of New Technologies, University of Warsaw, Banacha 2c, 02-097 Warsaw, Poland; j.miszkiewicz@cent.uw.edu.pl; 2 College of Inter-Faculty Individual Studies in Mathematics and Natural Sciences, University of Warsaw, Banacha 2c, 02-097 Warsaw, Poland

**Keywords:** antimicrobial peptides, cell-penetrating peptides, circular dichroism and fluorescence spectroscopy, secondary structure of peptides, membrane mimics

## Abstract

Many peptides interact with biological membranes, but elucidating these interactions is challenging because cellular membranes are complex and peptides are structurally flexible. To contribute to understanding how the membrane-active peptides behave near the membranes, we investigated peptide structural changes in different lipid surroundings. We focused on two antimicrobial peptides, anoplin and W-MreB_1–9_, and one cell-penetrating peptide, (KFF)_3_K. Firstly, by using circular dichroism spectroscopy, we determined the secondary structures of these peptides when interacting with micelles, liposomes, *E. coli* lipopolysaccharides, and live *E. coli* bacteria. The peptides were disordered in the buffer, but anoplin and W-MreB_1–9_ displayed lipid-induced helicity. Yet, structural changes of the peptide depended on the composition and concentration of the membranes. Secondly, we quantified the destructive activity of peptides against liposomes by monitoring the release of a fluorescent dye (calcein) from the liposomes treated with peptides. We observed that only for anoplin and W-MreB_1–9_ calcein leakage from liposomes depended on the peptide concentration. Thirdly, bacterial growth inhibition assays showed that peptide conformational changes, evoked by the lipid environments, do not directly correlate with the antimicrobial activity of the peptides. However, understanding the relation between peptide structural properties, mechanisms of membrane disruption, and their biological activities can guide the design of membrane-active peptides.

## 1. Introduction

The fight against pathogens resistant to antibiotics remains one of the most challenging problems of modern medicine. Since bacterial infections have become difficult to control [1] the search for new antibacterial drugs is a must. The breakthrough may be to design novel membrane-active peptides used alone or as antibiotic adjuvants [2,3]. The mechanism of action of such peptides is primarily based on their electrostatic interactions with the microorganism’s cell membrane. Peptides disrupt the cellular membranes, which changes their pH, and disturbs the osmotic balance and respiratory processes, leading to destruction of the bacterial cell [3]. 

Our study focusses on peptides interacting with different membrane models and membranes, including bacterial ones. We consider two types of peptides: cell-penetrating peptides (CPPs) and antimicrobial peptides (AMPs) [4]. Although these peptides have different biological functions, both interact with the cell membrane. For non-therapeutic CPPs, these interactions serve to transport the drug into bacteria. Contrary, interactions of most AMPs with the membrane result in membrane disintegration and damage, which inhibits cellular functions and destructs the cell. Thus, understanding how CPPs and AMPs interact with the membranes is crucial to design CPP-based carriers and AMP-based antibacterials. CPPs, apart from antibiotic-carriers, may also serve as antibiotic potentiators because by slightly disintegrating the membrane CPPs decrease the minimal inhibitory concentrations of the antibiotic necessary to inhibit bacterial growth. An example is the conjugate of the penetratin analog (one of the CPPs) with tobramycin, developed by Schmidt et al. [5,6]. This conjugate destabilizes bacterial membrane and inhibits protein synthesis showing 4 to 6 times stronger bactericidal activity against *E. coli* and *S. aureus* persister cells than tobramycin alone [5]. Another example of CPP use is a hybrid of a P14LRR peptide with the aminoglycoside antibiotic kanamycin [7]. These and other examples [2] show that synergy between a CPP and antibiotic may identify effective antibacterial lead compound.

How the peptides interact with the membranes depends on the sequence, structure and concentration of the peptide and composition of the membrane [8]. We selected membrane-active CPPs and AMPs, focusing on peptides that most probably adopt the active structure while surrounded by lipids. These peptides are partly unstructured in the buffer solution, but after binding to the membrane they are believed to adopt regular secondary structures, which have characteristic, distinguishable circular dichroism (CD) spectra [9,10]. 

Peptides that take on an active structure only after binding to the membrane are of particular interest because this transition is key to their biological function. Yet, the transition is a complicated process that depends not only on the peptide physicochemical properties, but also on the properties of the solvents. The propensity of peptides to form a well-defined secondary structure in water is relatively low because the intramolecular hydrogen bonds in the peptide compete with hydrogen bonds formed with solvent [9]. However, the potential to form secondary structures often increases in hydrophobic environments, such as organic solvents. Thus, binding of peptides or proteins to biological membranes is often accompanied by a conformational transition from a random coil to an α-helix [9].

Our aim was to determine how external lipid environment affects the secondary structures of membrane-active peptides. The peptides were selected based on the literature and database of AMPs [11] from natural and laboratory sources. We investigated three peptides: one with known cell-penetrating properties—(KFF)_3_K, and two with antimicrobial properties—W-MreB_1–9_ and anoplin. Peptide sequences, their net charges and hydrophobicity are shown in Table 1.

The (KFF)_3_K peptide is a popular CPP used to effectively transport peptide nucleic acid oligomers into both gram-negative and gram-positive bacteria [13,14,15,16,17,18]. Vaara and Porro [19] suggest that this synthetic peptide disrupts bacterial outer membrane. However, the structural changes of (KFF)_3_K occurring upon its interaction with bacterial membranes have not been studied so far.

The W-MreB_1–9_ AMP is the N-terminal fragment of the bacterial cytoskeletal protein MreB. The MreB proteins polymerize to form filaments and are responsible for the rod-shape of bacteria. The N-terminal amphipathic helix of MreB in *E. coli* is enough to bind this protein to a bacterial membrane [20]. Saikia et al. [21,22] used this N-terminal fragment of MreB as an antimicrobial peptide showing broad-spectrum activity against gram-negative and gram-positive bacteria as well as fungi. Interestingly, adding a Trp residue to the N-terminus of the 9-residue MreB_1–9_ induces a 3-fold better antimicrobial activity. According to Saikia et al. [21], the antibacterial activity of this peptide results from membrane damage as W-MreB_1–9_ destabilizes the bacterial membrane. Such enhanced peptide activity after adding Trp was explained by Trp interactions with the surface of the bacterial cell [23]. Shang et al. [23] showed that peptides containing Trp interact strongly with the external lipopolysaccharides (LPS) of gram-negative bacteria, and observed the highest bactericidal activity for peptides with Trp residues at the N-terminus. Based on these data, we used the MreB_1–9_ peptide with Trp attached at its N-terminus (Table 1) [21].

Finally, we selected anoplin, the simplest short helical AMP isolated from the venom of a solitary wasp *Anoplius samariensis* [24,25]. This peptide is active against gram-negative and gram-positive bacteria and is not hemolytic [24,26,27]. Anoplin is a decapeptide, amidated at the C-terminus, which interacts with the bacterial membrane (Table 1). In water, it is unstructured, but in sodium dodecyl sulfate (SDS) and trifluoroethanol buffers, as well as upon binding to a bacterial membrane, anoplin adopts a helical conformation [24,28,29]. Moreover, the structure of anoplin in the dodecylphosphocholine (DPC) micelles resolved by nuclear magnetic resonance spectroscopy [26] shows a helical conformation of this peptide (Figure S1).

Generally, the selectivity of peptides towards different membranes depends on the chemical composition of the membranes, which differs among bacteria [8,30,31]. Positively charged peptides interact with high affinity with the membranes containing negatively charged lipids, while their binding to neutral membranes is weak [31]. Eukaryotic membranes contain higher concentrations of heterogeneous phospholipids than membranes of prokaryotic organisms, such as bacteria with high content of negatively charged phospholipids [3,31]. Biological membranes are complex, so most studies of the peptide-membrane interactions are performed using membrane models [8,31,32]. The advantage of using simplified membrane models is that the influence of each lipid can be tested separately. The typical membrane models used to study the interaction between the peptides and membranes are detergent micelles and liposomes [4]. 

**Figure 1:**
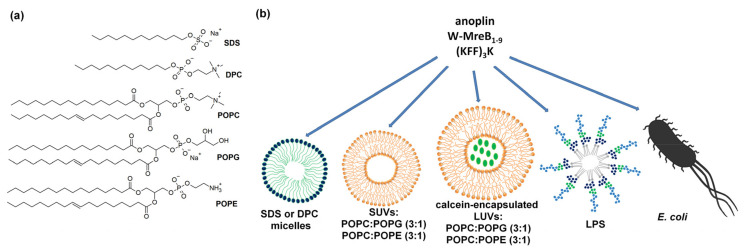
(**a**) Chemical structures of SDS, DPC, POPC, POPG and POPE. (**b**) Schematic representation of membrane mimics used in this work.

In this work, using CD and fluorescence spectroscopy, we investigated the interactions of three peptides with different cell membrane mimics, focusing on peptide conformational changes induced by the membrane. First, we used spherical SDS and DPC detergent micelles whose hydrophilic faces are directed towards water and hydrophobic tails towards the micelle center. The SDS micelles are negatively charged, making them good models of the prokaryotic membranes. Contrary, the DPC micelles are zwitterionic (neutral), making them good models of the eukaryotic membranes (Figure 1) [31,33].

Second, we used small unilamellar vesicles (SUVs) composed of 2-Oleoyl-1-palmitoyl-*sn*-glycero-3-phosphocholine (POPC), 2-Oleoyl-1-palmitoyl-*sn*-glycero-3-phospho-*rac*-1-glycerol (POPG), and 2-Oleoyl-1-palmitoyl-*sn*-glycero-3-phosphoethanolamine (POPE) lipids. POPG lipids are negatively charged, and POPC and POPE are neutral. POPE differs from POPC by the absence of three methyl groups at the nitrogen atom (Figure 1) [34]. The POPC:POPE or POPC:POPG mixtures, in a molar ratio of 3:1, form SUV liposomes making them resemble, respectively, eukaryotic and prokaryotic membranes [8].

However, biological membranes are more complicated than micelles or liposomes and contain various phospholipids, sterols, proteins and other molecules [35]. A key component of the outer membrane of gram-negative bacteria is LPS [30,35], which forms a negatively charged, semi-permeable barrier with composition depending on bacterial strain. LPS modulates the transport of antibiotics and the activity of AMPs and CPPs. Only few studies have been conducted to determine how the peptides interact with LPS of bacterial membranes [30,32] or live bacterial cells [32,36,37,38]. Thus, it is not yet clear what exactly happens once peptides get near bacterial cells. Therefore, inspired by the work of Avitabile et al. [32], apart from micelles and SUVs, we also performed experiments using LPS isolated from *E. coli* O111:B4 and live *E. coli* BL21(DE3) cells (Figure 1) [39]. 

Further, we investigated if structural changes of the peptides correlate with their biological activities. First, we determined to what extent the peptides affect membrane disruption by monitoring the release of calcein from large unilamellar vesicles (LUVs) (Figure 1). Monitoring the change of calcein fluorescence exerted by membrane-active peptides is a well-documented technique [31,40,41,42,43]. Second, we examined the antibacterial activities of the peptides by determining their minimum inhibitory concentrations (MIC) on two *E. coli* strains: K12 [44]—the wild type laboratory strain and BL21(DE3)—a strain deficient in the outer-membrane protease OmpT [39]. 

## 2. Results

### 2.1. Synthesis and Characterization of the Peptides

Anoplin, W-MreB_1–9_ and (KFF)_3_K were synthesized on solid-phase using the Fmoc method [45] and products were confirmed by mass spectrometry (MS, see Methods). The peptides were further purified by semi-preparative reverse phase high-performance liquid chromatography (RP-HPLC) and their purity >98% was verified by analytical RP-HPLC and MS (Figures S2–S4). Table 1 shows peptide physicochemical properties, as well as their theoretical and measured molecular weights and retention times. 

### 2.2. CD Spectra of Anoplin

**Figure 2:**
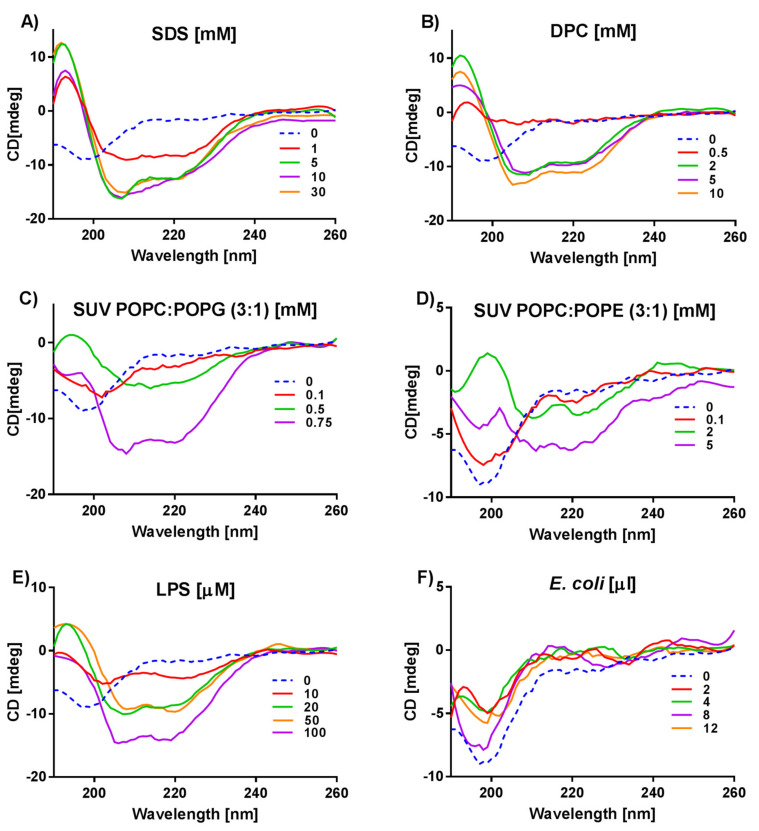
CD spectra of anoplin recorded in the presence of (**A**) SDS micelles, (**B**) DPC micelles, (**C**) POPC:POPG (3:1) SUVs, (**D**) POPC:POPE (3:1) SUVs, (**E**) LPS, (**F**) *E. coli* BL21(DE3).

Figure 2 shows the CD spectra of anoplin in the phosphate buffer and membrane mimics. In the phosphate buffer, anoplin adopts a random coil as evidenced by the negative band close to 200 nm (blue dashed line in Figure 2), which agrees with previous reports [24,28,29]. However, in the SDS and DPC detergents (Figure 2A,B), at concentrations above the critical micelle concentration (CMC), the negative bands at 209 and 222 nm indicate an α-helix. These changes suggest that anoplin interactions with either SDS or DPC micelles lead to structural changes because the pure buffer solution preserves the random coil. Further, after the SDS or DPC concentration is increased, the shapes and intensities of the CD spectra remain similar meaning that, once adopted, the anoplin helical conformation does not further depend on the micelle concentration. 

Introducing anoplin into anionic or zwitterionic liposomes (Figure 2C,D) changes the CD spectra of this peptide as compared to the spectra obtained in the buffer or micelles. Although at 0.75 mM of POPC:POPG and 2 mM of POPC:POPE SUVs, two helix-indicating negative bands at 209 and 220–222 nm are present (Figure 2C,D), the positive band at 195–197 nm is not visible. This suggests the presence of different peptide structures. In addition, anoplin interacts with the liposomes in a concentration dependent manner. To better understand what happens with anoplin in this environment, we increased lipid concentrations (Figure S5). Figure S5 suggests a “critical concentration” of liposomes in which the peptide helical conformation reaches an equilibrium state. This concentration is 0.75 mM for POPC:POPG and about 5 mM for POPC:POPE SUVs. Above these concentrations, the CD signal from anoplin disappears with increasing lipids concentration. This could mean that anoplin helical structure reached conformational equilibrium at a “critical point” of lipid concentrations.

The CD spectra of anoplin in the presence of LPS (Figure 2E) are similar to its spectra obtained in POPC:POPG SUVs, i.e., mimicking prokaryotic membranes. For 20 µM LPS, anoplin spectra show two negative bands characteristic for an α-helix (Figure 2E, green line). As the LPS concentration increases up to 100 µM, the CD signal intensity also increases. Again, similar as for anoplin in SUVs, this 100 µM LPS concentration point seems to be the optimal one for anoplin-LPS interactions.

In the presence of live bacteria (Figure 2F), the CD spectra of anoplin suggest a random coil, the same as in the buffer. However, spectral intensities in the presence of *E. coli* are slightly lower than the intensity of anoplin spectrum in the buffer. 

### 2.3. CD Spectra of W-MreB_1–9_

**Figure 3:**
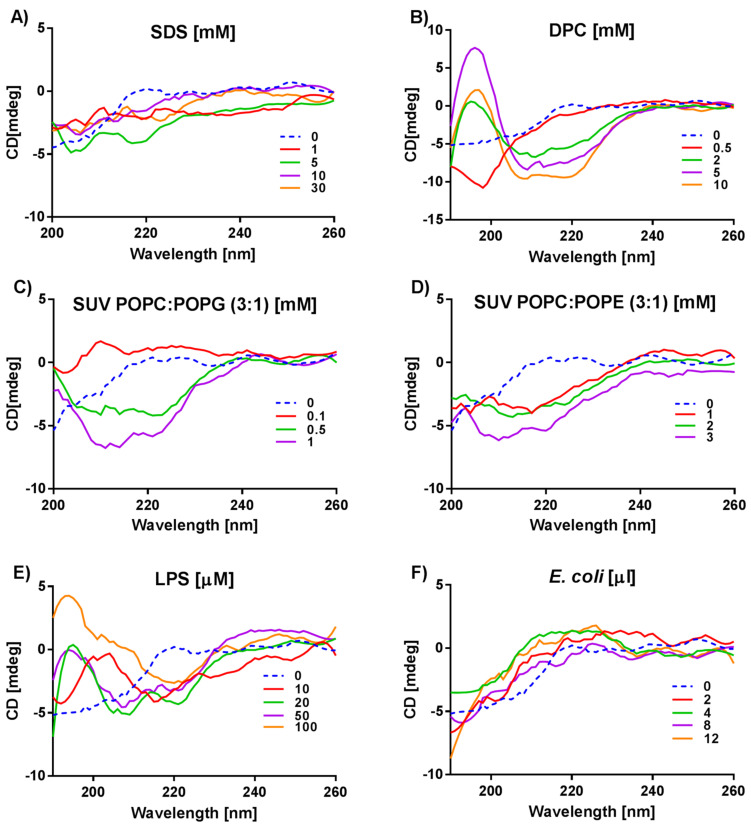
CD spectra of the W-MreB*_1–9_* studied in different membrane environments: (**A**) SDS micelles, (**B**) DPC micelles, (**C**) POPC:POPG (3:1) SUVs, (**D**) POPC:POPE (3:1) SUVs, (**E**) LPS, (**F**) *E. coli* BL21(DE3).

The CD spectroscopy of the W-MreB_1–9_ peptide indicates that it is disordered in buffer solution (blue dashed line in Figure 3). In the presence of SDS, the peptide changes its conformation only slightly (Figure 3A). Contrary, the structure of W-MreB_1–9_ surrounded by DPC micelles changes more drastically, as evidenced by intensive negative bands appearing at about 209 and 222 nm at a DPC concentration of 2 mM and higher (Figure 3B). These spectra indicate an α-helix structure of the peptide in the DPC micelles.

The shapes of W-MreB_1–9_ CD spectra in the presence of SUVs are presented in Figure 3C,D. Due to high HT (high tension) values detected below 200 nm during CD measurements of W-MreB_1–9_ in SDS and SUVs, for these lipids we present the CD spectra only in the range 200–260 nm. This makes it difficult to interpret these spectra because it is not possible to confirm the presence of the characteristic positive band for the α-helix or β-sheet at about 195 nm. Nevertheless, the characteristic negative bands are visible, such as those at 1 mM POPC:POPG and 3 mM POPC:POPE that indicate a helix. 

Unexpectedly, for the W-MreB_1–9_ peptide, we observe completely different CD spectra in LPS (Figure 3E) than in the presence of live *E. coli* (Figure 3F). At LPS concentrations of 20 and 50 µM, the negative bands at about 209 and 220–222 nm are visible, indicating the presence of an α-helix. When the concentration of LPS in the peptide solution increases to 100 µM, the negative band at 209 nm disappears. The changing shape of the CD spectra suggests a change in the peptide conformation. We suppose that with an increase in LPS concentration, the amount of the α-helix decreases and a β-sheet appears. Contrary, in *E. coli* the shapes of the W-MreB_1–9_ spectra suggest unordered peptide structures in all cell concentrations, similar as for anoplin. 

### 2.4. CD Spectra of (KFF)_3_K

**Figure 4:**
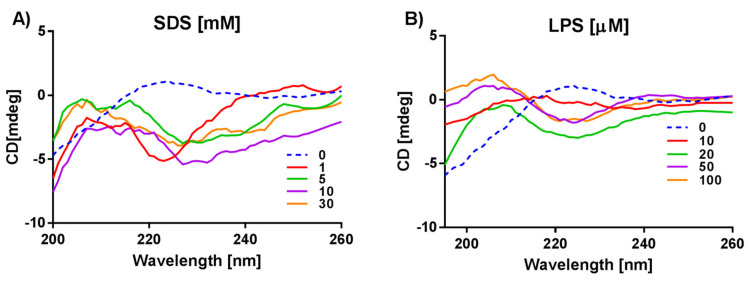
CD spectra of the (KFF)_3_K studied in different membrane environments: (**A**) SDS micelles, (**B**) LPS.

The CD spectra of (KFF)_3_K in the buffer solution show unordered peptide structure (blue dashed line in the Figure 4 and Figure S6). In SDS micelles, at low SDS concentration of 1 mM a low intensity negative band suggests a β-sheet structure of (KFF)_3_K (Figure 4A). However, again monitoring the (KFF)_3_K CD spectra in the presence of SDS and DPC resulted in high HT values below 200 nm, so the spectra in Figure 4A and Figure S6A can be interpreted only in the range 200–260 nm. In the presence of DPC micelles, SUVs and bacteria (Figure S6), the (KFF)_3_K spectra are similarly irregular and no characteristic secondary structure can be assigned suggesting either unordered peptide or a mixture of structures.

The (KFF)_3_K spectra in LPS show a negative band in the range 220–230 nm and a positive band in the range 200–215 nm (Figure 4B). The shapes of the (KFF)_3_K CD spectra in LPS suggest the presence of β-sheet and β-turn structures.

### 2.5. Analysis of the Helicity of Peptides Based on CD Spectra

The helicity is easiest to examine from the mean residue ellipticity [*θ*] at the 222 nm wavelength (Equation (1)) [25,28,46]. The percentages of helicity in the peptide structures, estimated based on the [θ]_222_ nm values (Equation (2)), are shown in Table 2.

Anoplin is more helical if placed in SDS micelles (up to 71% helicity) or DPC micelles (up to 63%) than in the buffer solution (only up to 9%), which agrees with previous reports [24,27,28,29]. In the POPC:POPG solution, the maximum anoplin helicity (72%) is reached at 0.75 mM SUVs. Contrary, for POPC:POPE SUVs the maximum helicity of anoplin is not higher than 32%. For anoplin placed in LPS, similarly as in POPC:POPG SUVs, the maximum 73% helicity occurs for LPS concentration of 100 µM. The helicity of anoplin is negligible in the presence of in live *E. coli* cells.

Both W-MreB_1–9_ and (KFF)_3_K in the phosphate buffer show positive ellipticity at 222 nm, so it was impossible to determine any percentage of helicity from these spectra. The W-MreB_1–9_ peptide helicity does not exceed 50%, regardless of the membrane mimic, and ranges from 6% to 18% in SDS and from 5% to 48% in DPC, indicating that the presence of zwitterionic micelles increases the helicity of this peptide. Contrary, for W-MreB_1–9_, the helicity is overall higher in zwitterionic SUVs than in negatively charged SUVs. The percentage of W-MreB_1–9_ helicity in the presence of LPS is in the range 15–24% for all LPS concentrations. The helicity of (KFF)_3_K ranges from 13% to 29% in SDS and from 4% to 12% in DPC. The percentage of helicity of W-MreB_1–9_ in the presence of bacteria and (KFF)_3_K in SUVs and bacteria could not be determined.

To compare the helicity calculated based on the [θ]_222_ nm values (Equations (1) and (2), Table 2) with other methods that estimate the secondary structure content in peptides from their CD spectra, we used the DichroWeb server [47,48]. The secondary structure contents calculated using various algorithms and reference data sets expressed as fractions of α-helices, β-sheets, turns and unordered structures are gathered in Figure S7 and Table S1 for anoplin, Figure S7 and Table S2 for W-MreB_1–9_, and Figure S7 and Table S3 for (KFF)_3_K. The best fit, with the lowest normalized root mean square deviation (NRMSD), was obtained with the CDSSTR method (Figure S7). For all peptides and in all solutions the results show mixtures of different peptide conformers. 

### 2.6. Calcein Leakage from LUVs 

**Figure 5:**
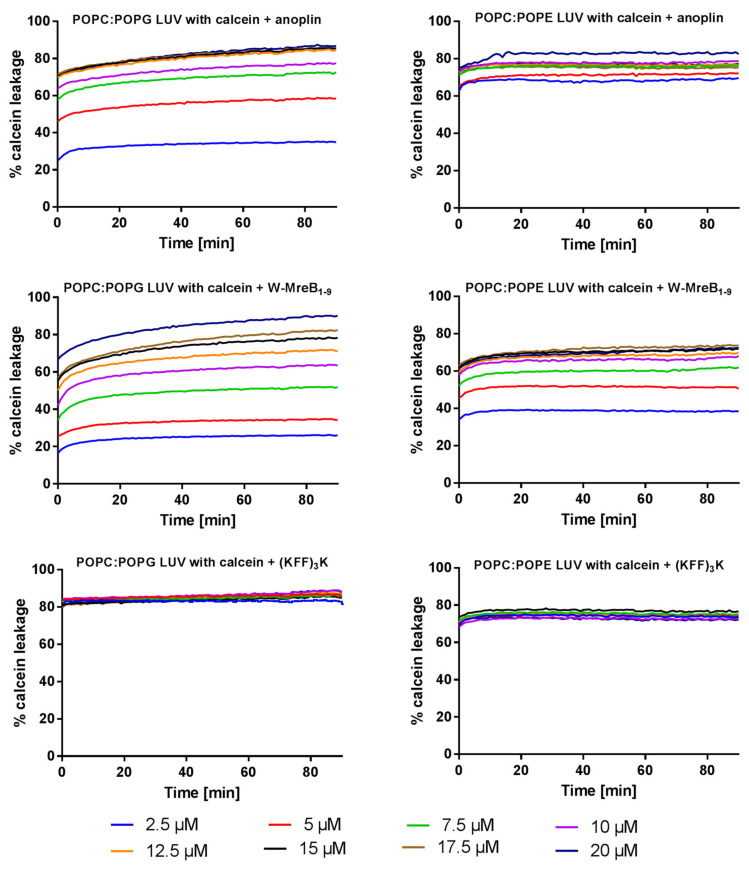
The percentages of calcein leakage from the negatively charged POPC:POPG (3:1) LUVs (left plots) and neutral zwitterionic POPC:POPE (3:1) (right plots) in the presence of different concentrations of anoplin, W-MreB_1–9_, and the (KFF)_3_K peptide. The plots show averages from three experiments.

To quantify the destructive effect of peptides on the membranes, we investigated if peptides induce liposomal disorders by monitoring calcein release from two types of calcein-loaded LUVs. We used the same liposome composition and lipid ratios as for the CD experiments with SUVs, namely POPC:POPG (3:1) and POPC:POPE (3:1) (Figure 1). The leakage of calcein from LUVs (without the peptides) is negligible during the time course of the experiment. However, the exposure of liposomes to peptides causes the trapped calcein to rapidly leak. The observed percentages of calcein leakage dependent on the peptide concentrations are shown in Figure 5.

In all experiments, after adding the peptide to calcein-loaded LUVs fluorescence increases, which further allows calculating the percentages of calcein leakage. Anoplin, already at a concentration of 12.5 μM and within 10 minutes, induces 74% of calcein leakage from the POPC:POPG LUVs. The maximum, anoplin-induced calcein leakage from the POPC:POPG LUVs is about 87% and from POPC:POPE LUVs—about 83%. However, the leakage of the dye from the zwitterionic POPC:POPE liposomes is faster at lower anoplin concentrations. Already after 10 min of incubation with 5 μM anoplin, 70% of calcein leaks out of POPC:POPE LUVs. For charged POPC:POPG LUVs the amounts of leaked calcein depend on anoplin concentration.

Also, for W-MreB_1–9_ and both LUV types, calcein leakage depends on the peptide concentration. After adding W-MreB_1–9_, calcein leaks slower, but in the end to a higher extent, from POPC:POPG LUVs than from POPC:POPE LUVs. The maximum calcein leakage from POPC:POPG LUVs is about 90% and from POPC:POPE LUVs—74%. 

Surprisingly, the plots of calcein leakage in the presence of (KFF)_3_K show that, for both LUV types, the leakage does not depend on peptide concentration. Calcein leakage from both LUVs is similarly high and occurs already at 2.5 µM (KFF)_3_K, i.e., the lowest tested peptide concentration.

### 2.7. Antimicrobial Assays

To assess if the observed conformational changes of the peptides relate to their biological functions, we determined peptide antibacterial activities. Table 3 shows the MIC values assayed against gram-negative bacteria: *E. coli* K12 [44] and *E. coli* BL21(DE3) [39] strains and Figure S8 shows the optical densities. Conventional antibiotics, ampicillin and tetracycline, were used as positive controls (Figure S9). Overall, the peptides exhibit low activities (i.e., high MIC) against both *E. coli* strains. Anoplin and (KFF)_3_K have the same MICs for both strains, with the MIC for the K12 strain corresponding to the previously published numbers [13,16,28,49,50]. The highest determined MIC of 256 µM is for W-MreB_1–9_ and the *E. coli* BL21 strain, deficient in the outer membrane OmpT protease. Its MIC for the K12 strain is half this value, but the published minimum lethal concentration of this peptide (of 5 µM) against *E. coli* K12 was measured under different conditions than in our experiments (the bacteria were cultured in the phosphate buffer and not in the medium) [21], so we cannot compare these data with our results.

## 3. Discussion

The usefulness of CD spectroscopy for monitoring conformational changes in peptides using different membrane models has been well documented [8,31,32]. Inspired by these works, we investigated the structural changes of one CPP peptide ((KFF)_3_K) and two AMP-type peptides (anoplin and W-MreB_1–9_) in the presence of different membrane models, LPS, and *E. coli* cells. The simplest membrane models were single-layered detergents: charged SDS and neutral DPC micelles. The more complex models were SUVs consisting of either POPC:POPG (3:1) or POPC:POPE (3:1) lipids. These liposomes were designed to correspond to prokaryotic and eukaryotic membranes, respectively. The most complex, but at the same time most realistic models were the LPS isolated from *E. coli* O111:B4 and live *E. coli* BL21(DE3) cells [32].

In most membrane mimics, the peptide CD spectra changed shapes and their negative bands shifted indicating that while interacting with the membranes the peptides take on different conformations than in buffer solutions [8,31,32]. The share of the secondary structure elements in these peptides was estimated either by calculating the peptide helicity from the [θ]_222_ values or matching the experimental CD spectra with the reference spectra of already characterized proteins. For the latter, we used two DichroWeb algorithms, CDSSTR and CONTILL [47,48], which apply different methods of spectrum deconvolution. We used three different spectra reference sets: Set 4, Set 7 and SP180. Sets 4 and 7 are based on CD spectra of mainly soluble proteins, and the SP180 set contains also spectra characteristic for the membrane proteins [51,52]. Different algorithms and spectral reference sets were used to verify the results obtained with different methods. Unfortunately, the NRMSD, which quantifies the quality of the fit, obtained for our peptides depends on the algorithm and reference set (Tables S1–S3). The NRMSD error using the SP180 set was in most cases higher than for the other two sets. Thus, it is not possible to assess, which algorithm and database are most suitable to analyze the secondary structures of short peptides in the presence of membrane mimics. 

Based on the analyses from the Dichroweb server (Figure S7, Tables S1–S3) and the literature [43], we interpreted the CD data bearing in mind that the peptide spectrum may represent a combination of several different peptide conformers and relates only to the average structures. Therefore, interpretation of such CD spectra is difficult, for example, as in the case when the negative band corresponding to a β-sheet partly coincides with the two negative bands corresponding to an α-helix [9,10]. However, some CD spectra as the one of anoplin in SDS or DPC micelles (Figure 2A,B) or W-MreB_1–9_ in DPC (Figure 3B), suggest with no doubt α-helical conformations. Nevertheless, a more demanding task was to interpret the CD spectra of peptides obtained in the presence of more complicated membrane mimics, such as liposomes. The negative bands, characteristic of an α-helix, are less visible in the W-MreB_1–9_ spectra in SUVs than in the spectra recorded in the presence of DPC. Furthermore, at the same concentration, the intensity of the W-MreB_1–9_ spectrum in SUVs is higher than the intensity of the helical W-MreB_1–9_ in DPC and helical anoplin in SDS and DPC. This suggests that the W-MreB_1–9_ peptide is intrinsically flexible and can adopt a mixture of conformations (Figure 3). This also means that the α-helix in the W-MreB_1–9_ structure is reduced in favor of β-sheets, which is evidenced by the helicity calculated based on [θ]_222_ (Table 2). Previously, the structure of the W-MreB_1–9_ peptide in the presence of SUVs was investigated in the work of Saikia group [22]. Their CD experiments were carried out under different conditions than ours (different buffer, liposome composition and lipid ratios) and for only one lipid concentration, so we cannot compare with their results. However, for some derivatives of the W-MreB_1–9_ peptide, the authors observed a mixture of different conformations in the presence of liposomes. Rusell et al. [43] also write that CD spectra in the presence of liposomes may represent a combination of a number different peptide conformers because peptide-liposome interactions depend on the liposome type and concentration. Another CD spectrum difficult to interpret is the one of (KFF)_3_K in LPS, which shows positive bands that we assign to a β-turn (Figure 4). However, many types of turns are possible, and unfortunately they are poorly characterized in the literature [53,54]. Also, because of the large spectral variation of the β-sheet and β-turn structures, it is difficult to interpret the CD spectra containing these conformations.

The examined peptides are all positively charged (+4e for anoplin and W-MreB_1–9_, and +5e for (KFF)_3_K) and similarly hydrophobic (Table 1), but we found no relation between the peptide physicochemical properties and secondary structures they adopt in the membrane solutions. However, the peptides contain alternating hydrophobic and hydrophilic amino acids in the sequence, what could promote the formation of peptide dimers and oligomers through hydrophobic interactions between peptide side chains. Thus, oligomerization could be a factor affecting peptide interactions with the membranes [55,56,57]. 

Nevertheless, we found that peptide structural complexity depends on the charge of the membrane. Importantly, the peptides show different CD spectra in the presence of zwitterionic DPC and anionic SDS micelles, as well as zwitterionic POPC:POPE and anionic POPC:POPG liposomes. In addition, since LPS is a large, hydrophilic, and negatively charged molecule, the positively charged peptides can accumulate on the surface of a single-layer LPS construct [58,59]. For both anoplin (Figure 2) and W-MreB_1–9_ (Figure 3) we observe similarities in the CD spectra obtained in the presence of LPS and negatively charged liposomes. Thus, the overall charge of the membrane affects the conformations adopted by the peptides. 

Both the membrane charge and peptide concentrations determine peptide effectiveness in disrupting lipid structures. We observed this by monitoring the leakage of calcein from LUVs after incubation with peptides. The peptides are positively charged, so their long-range electrostatic interactions with the negatively charged liposomes are, in principle, more favorable than with zwitterionic liposomes. Indeed, for the highest concentrations of peptides, their final disruptive effect is the highest for the negatively charged POPC:POPG LUVs. Also, the peptide-induced disruption of liposomes depends on peptide concentration to a higher extent for POPC:POPG (at least for anoplin and W-MreB_1–9_) than for the neutral POPC:POPE LUVs.

The release of calcein was most effective and also independent of peptide concentration for (KFF)_3_K, which showed antibacterial activity comparable to anoplin (Table 3, Figure S8). Although (KFF)_3_K is typically considered a CPP and not AMP, its MIC of 32 µM is not surprising. The group of N. Patenge reported that (KFF)_3_K has an antibacterial effect against gram-positive *Streptococcus pyogenes* already at 5.6 µM [60]. Furthermore, Mohamed et al. [61] proved antibacterial activity of (KFF)_3_K and its strong synergistic effect with gentamicin and amikacin against methicillin-resistant and susceptible strains of *Staphylococcus pseudintermedius*. Based on [60,61] and our results showing the same antibacterial activity of (KFF)_3_K and anoplin, (KFF)_3_K should be regarded not only as a CPP, but also at higher concentrations as a potential antibacterial peptide. 

Peptide interactions with lipids are essential for the formulation of membrane pores necessary to exert antibacterial activities [31,43]. Although the mechanism of pore formation and membrane disruption may differ among the peptides, it depends on peptide structural changes evoked by peptide-membrane interactions. Indeed, in our CD experiments most peptides change conformations upon interacting with lipids. For example, we observed structural changes of anoplin in different concentrations of SUVs and LPS, though not in the presence of micelles (Figure 2B,C, Figure 5 and Figure S5). Crowding could be one of the contributing factors. In CD experiments performed with the constant peptide concentration, increasing the membrane concentration “crowds” the sample. Peptides have less space due to excluded volume effect, and probably start to accumulate on the surface of membrane mimics. Indeed, membrane active peptides, such as anoplin, were shown to accumulate on the liposome surface [42,62]. Anoplin binds to the liposome surface but at certain liposome concentration (called the “critical point”), anoplin secondary structure reaches an equilibrium. This process can be crucial for the destructive effect of peptides on cell membranes [31,43]. In our CD experiments with anoplin, the “critical points” of the lipids and LPS concentrations were estimated as 0.75 mM for POPC:POPG, 5 mM for POPC:POPE, and 100 µM for LPS. Even though the CD spectra recorded for samples with higher concentrations of lipids or LPS could be affected by light scattering effects, we tried to reduce this disturbance by adjusting the concentrations of peptides and the length of the optical path [10,31,32,63], as well as by simultaneously controlling the HT values during the CD scans. However, in some cases the interpretation of CD data at low wavelengths is limited.

Overall, it turned impossible to correlate the spectroscopic data obtained for the peptides in model membranes with peptide biological activity although it is clear that the type and charge of the membrane define how these peptides interact with the membranes. For example, we demonstrated that anoplin differently interacts with the anionic and zwitterionic membrane models. From a therapeutic point of view, this is a critical observation, which suggests that the positively charged anoplin preferentially interacts with the negatively charged bacterial cell membrane rather than with the eukaryotic cell membrane [8,31]. Surprisingly, the peptides adopt different secondary structures when they interact with micelles or SUVs than with the bacterial membrane. So the first step towards understanding the mechanisms governing the actions of membrane-active peptides is to determine their tendency to accumulate in the presence of a membrane.

Although we observed peptide conformational changes in LPS, it proved impossible to observe any structural changes in the presence of live bacteria. The differences between the CD spectra recorded in the presence of LPS and bacteria may be due to the different structure of the isolated LPS than the cell envelope. The isolated and purified LPS adopts the structure of a single-layer micelle, while the bacterial cell has a bilayer barrier with one external LPS layer. Thus, it looks like the membrane mimics that are typically used in experiments (micelles, liposomes, LPS) poorly imitate the outer-membrane of live *E. coli*. It could also be that the peptide structures observed in the micelles and SUVs become unstable in bacterial cultures, which would further explain the peptide low antibacterial activity. After all, in the experiments monitoring calcein release from LUVs, the peptides induced liposome disruption, which means that peptide structures showing in the CD spectra in the corresponding SUVs are indeed the active structures. These observations suggest that stabilization of active peptide structures observed in the liposomes may improve peptide activity.

## 4. Materials and Methods 

SDS, DPC, LPS, POPC, POPG and POPE were purchased from Sigma Aldrich. All chemicals were of analytical or reagent grade. All buffers were prepared using distilled water.

### 4.1. Peptide Synthesis and Purification

Peptides were synthesized using the solid-phase peptide synthesis (SPPS) method and the standard Fmoc/t-Bu chemistry [45], on a 100 μmol scale and Rink-amide resin (TentaGel S RAM resin, amine groups loading of 240 μmol/g; this resin has a linker which yields a C-terminal amide upon trifluoroacetic acid cleavage of the peptide). Fmoc-protected amino acids were assembled as active derivatives in a 3-fold molar excess, using HATU with 1-hydroxy-7-azabenzotriazole (HOAt) and collidine (1:1:2), and the dimethylformamide/N-methylpyrrolidone (1:1, *v*/*v*) solution-coupling method for 1.5 h. The Fmoc deprotection was accomplished using 20% piperidine in dimethylformamide for 2 cycles (5 and 15 min). The reaction progress of each step was confirmed by the negative result of the Kaiser test [64]. The removal of protecting groups from amino acids and cleavage of peptides from the resin were achieved by treatment with trifluoroacetic acid/triisopropylsilane/m-cresol (95:2.5:2.5; *v*/*v*/*v*) mixture for 60 min. The obtained crude oligomers were lyophilized and purified by reversed phase chromatography (RP-HPLC). Analytical and semi-preparative RP-HPLC of the peptides were performed on the Knauer C18 columns (4.6 × 250 mm, 5 μm particle size and 8 × 250 mm, 5 μm particle size, respectively). The SYKAM system was applied, and the mobile phase gradient profile was as follows: from 20% to 80% in 30 min for anoplin, from 25% to 55% in 30 min for W-MreB_1–9_, from 0% to 50% in 30 min for (KFF)_3_K with buffer A (0.1% trifluoroacetic acid in acetonitrile) and buffer B (0.1% trifluoroacetic acid in water) at a flow rate of 1.5 mL/min, λ = 220 nm, (Figures S2–S4). The presence of peptides was confirmed by MS using the Q-TOF Premier mass spectrometer (Figures S2–S4). Purified peptides were obtained as salts with a trifluoroacetate anion. To exchange the anion to hydrochloride before the spectral measurements, peptides were dissolved in a 0.1 M HCl solution, frozen, and lyophilized.

### 4.2. Preparation of Micelles, POPC:POPE and POPC:POPG SUVs

Stock solutions with the 250 mM SDS and 200 mM DPC, i.e., the concentrations higher than their CMC (for SDS CMC = 4.5 mM, and for DPC, CMC = 1.1 mM [65,66]), were used as micelle solutions. Appropriate amounts of SDS and DPC were dissolved in a 10 mM phosphate buffer, pH 7.0 and vortexed. Prepared solutions were diluted in a cuvette directly before the measurement. Final concentrations of SDS used for CD studies were 1 mM, 5 mM, 10 mM, 30 mM and final concentrations of DPC were 0.5 mM, 2 mM, 5 mM, 10 mM.

The liposomes were prepared using standard procedures [8,31,42,67]. Appropriate amounts of dry 3:1 POPC:POPG and 3:1 POPC:POPE (mol/mol) were weighed to yield the final lipid concentration of 50 mM. The lipids were dissolved in chloroform and vortexed for 3 min. The sample was dried under nitrogen, dissolved in dichloromethane, and next was dried overnight under high vacuum. The lipids were hydrated with 2 mL of buffer (10 mM sodium phosphate, pH 7.0) and vortexed extensively. SUVs were prepared by sonication of the milky lipid suspension using a titanium tip ultra-sonicator (TEFIC ultrasonic homogenizer sonicator model TF-150N) for approximately 40 min in an ice bath until the solution became transparent. The titanium debris was removed by centrifugation at 8,800 rpm for 10 min using an Eppendorf table top centrifuge. Final concentrations of lipids used for CD studies were within the range 0.1–5 mM.

The LPS isolated from *E. coli* O111:B4 was dissolved in 10 mM phosphate buffer, pH 7, at a 50 µM concentration and before use it was subjected to temperature cycles between 4° and 70 °C, interrupted by vortexing [68]. Different amounts of LPS solution were added to peptide solutions, and CD spectra were recorded. Final concentrations of LPS used were 10 µM, 20 µM, 50 µM and 100 µM.

### 4.3. Preparation of Cell Culture for CD Experiments

The *E. coli* BL21(DE3) strain [32] was used. To prepare inocula, bacteria were grown overnight in LB at 37 °C with shaking (600 rpm). The refreshed culture in lysogeny broth (LB) was brought to OD_600_ of 0.6–0.8, centrifuged (5000 rpm, 10 min, 4 °C) and resuspended in 10 mM phosphate buffer, pH 7.0, to OD_600_ of 1. The peptides were dissolved in 10 mM phosphate buffer, pH 7.0, at 60 µM concentration. Different amounts of cells (2 µL, 4 µL, 8 µL, 12 µL) were added to peptide solutions and CD spectra were recorded (the final sample volume for each measurement was 100 µL).

### 4.4. Recording of CD Spectra 

CD spectra were recorded in: aqueous buffer solution (10 mM phosphate buffer, pH 7.0), in the presence of varying concentrations of SDS or DPC micelles, POPC:POPG (3:1) or POPC:POPE (3:1) phospholipids, LPS, and live *E. coli* bacteria. In all CD experiments, peptide concentrations were 60 μM. The spectra were collected using the Biokine MOS-450/AF-CD spectrometer equipped with the Xe lamp using a 0.1 cm CD cell. The acquisition duration time was 2 s with a resolution of 1 nm. The measurements were performed in 10 mM phosphate buffer, pH 7, in the wavelength range 190–260 nm and at room temperature. The graphs presenting the CD spectra were smoothed with the Savitzky-Golay method and presented using GraphPad. Contributions due to the micelles, mixed SUVs, LPS and bacteria, were eliminated by subtracting their spectra from the corresponding peptide + micelles, mixed SUVs, LPS and bacteria mixtures. Data for W-MreB_1–9_ in SDS, DPC and SUVs and (KFF)_3_K in SDS and DPC below 200 nm were not taken for analysis because their recording produced a HT value over 600 V giving a low signal-to-noise ratio. The presented CD spectra are the averages of three scans. Each CD experiment was conducted twice to confirm the repeatability of the spectra.

### 4.5. Secondary Structure Analyses of CD Data

The CD curves were used to calculate the percentage of α-helix in each peptide from the observed molar ellipticity at 222 nm ([θ]_222_). The mean residue ellipticity [θ] (deg*cm^2^/dmol) was calculated using the relationship:

(1) $${\left[\theta \right]}_{222}=\frac{\left(100\;\cdot \;\theta_{obs}\right)}{l\;\cdot \;c\;\cdot \;n}$$

where *θ_obs_* is the measured ellipticity in mdeg, l is the path length in cm, c is concentration of the peptide in mM, and n is the number of peptide residues [24,28,29,46].

The percentage of α-helix was estimated based on the [θ]_222_ values according to the Chen equation [46]:

(2) $$\%\;\alpha –helix=100\;\cdot \;\frac{{\left[\theta \right]}_{222}}{-{\left[\theta \right]}^{\infty }\;\cdot \;(1-\frac{k}{n})}$$

where the value [θ]^∞^, which corresponds to the 100% helicity at 222 nm, is estimated as 39,500, and the constant *k* is equal to 2.57 [25,28,46].

To assess the content of different secondary structures from CD spectra, the DichroWeb [47,48,51] software was used. The spectra were analyzed with the CONTINLL and CDSSTR analysis programs and three different data sets: DataSet4, DataSet7 and SMP180 [51,52]. 

### 4.6. Preparation of Calcein-Encapsulated LUVs

The calcein-encapsulated LUVs were prepared using a previous method [31,40,42,43,69]. The LUV composition used in the fluorescence assay (see below) was the same as the SUV composition in the CD experiments. This approach allowed comparing the results of these experiments and correlate the destructive activity of peptides on liposomes with their structure. The negatively charged lipids composed of POPC:POPG (3:1) and zwitterionic lipids composed POPC:POPE (3:1) were dissolved in chloroform, dried with a stream of argon, dissolved in DCM, and again dried with argon and under pressure using a rotator (for 3 h). The lipid layer was resuspended in dye buffer solution (70 mM calcein, 10 mM Tris, 150 mM NaCl, and 0.1 mM EDTA, pH 7.4). The suspension was extruded 21 times through polycarbonate filters (two stacked 100-nm pore size filters) with an Avanti Polar Lipid extruder. The untrapped calcein was removed from the solution by gel filtration on a Sephadex G-75 column. The lipid concentration was calculated from the weighed, starting lipid mass and the final dilution of the calcin-loaded LUV fraction from the column. 

### 4.7. Calcein Dye Leakage Assays

Aliquots of the liposome suspensions were diluted using Tris-HCl buffer to a final concentration of 2.2 mM. Peptide solutions with concentrations ranging from 2.5 to 20 µM were added to the liposomes. The leakage of calcein from LUVs was monitored by measuring the fluorescence intensity at the excitation wavelength of 490 nm and emission wavelength of 520 nm on a microplate reader from BioTek (Winooski, VT, USA) using Corning® Thermowell PCR 96 well plates. The measurement was conducted for 90 min at 25 °C, and the scan was performed every 1 minute. The maximum dye leakage release was obtained using 10% Triton X-100. The percentage of the calcein release caused by the peptides was calculated using the following equation [31,40,42,43,69]:

(3) $$\%\;\mathrm{calcein}\;\mathrm{leakage}=\frac{\left(F-F_{0}\right)}{\left(F_{100}-F_{0}\right)}\;\cdot 100\%$$

where *F*_0_ is the fluorescence intensity of the liposomes (background), and *F* and *F*_100_ are the fluorescence intensities of the peptides and Triton X-100, respectively. All experiments were performed in triplicates and the results are reported as the averages.

### 4.8. Bacterial Growth Inhibition

The MICs of (KFF)_3_K, anoplin, W-MreB_1–9_, and antibiotics were determined by broth microdilution according to Clinical and Laboratory Standards method M07-A10 (CLSI, 2015). The MIC was established as the concentration that prevented visibly detectable bacterial growth after 20 h of treatment. The *E. coli* K-12 and BL21(DE3) strains were cultured in LA, and next in the LB medium at 37 °C with shaking. For susceptibility tests, bacteria were grown in a cation-adjusted Mueller Hinton Broth at 37 °C with shaking to the exponential phase and diluted to ~5 × 10^5^ CFU/mL. Suspended cells were added to the wells of sterile 96-well plates containing peptides and antibiotics in different concentrations (256, 128, 64, 32, 16, 8, 4, 2, 1, 0.5 µM). Then, plates were incubated at 37 °C in a microplate reader for 20 h. The optical density at 600 nm (OD_600_) of the dilutions was measured in 10-min intervals. Measurements were preceded by a brief shaking of the plates to suspend bacterial cells. Each MIC experiment for the peptides was performed in at least four independent biological replicates.

## Figures and Tables

**Table 1:** Peptide sequences, net charge at pH 7.4, mean hydrophobicity (H, predicted by Heliquest [12]), calculated and detected molecular weight (MW), and retention times (RT) from RP-HPLC analysis; all peptides have an amide at the C-terminus.

Peptide	Sequence (N->C)	Net Charge	H	MW (g/mol)		RT (min)
				Calculated	Detected	
anoplin	GLLKRIKTLL	+4	0.587	1153.5	1153.8	11.3
W-MreB_1–9_	WMLKKFRGMF	+4	0.700	1342.7	1342.5	11.1
(KFF)_3_K	KFFKFFKFFK	+5	0.678	1525.1	1524.8	24.1

**Table 2:** Percentage of helicity in the peptides calculated based of the [*θ*]_222_ values (Equations (2)). For positive [*θ*]_222_ values, the % helix was not determined because of its negative value.

MembraneMimic		% Helix		
		Anoplin	W-MreB_1–9_	(KFF)_3_K
buffer		9	-	-
SDS [mM]	1	48	8	29
	5	68	18	13
	10	68	6	20
	30	71	13	18
DPC [mM]	0.5	8	5	12
	2	52	27	-
	5	53	38	4
	10	63	48	8
POPC:POPG (3:1) [mM]	0.1	16	3	-
	0.5	30	29	-
	0.75	72	-	-
	1	-	27	-
POPC:POPE (3:1) [mM]	0.1	27	-	-
	1	-	10	-
	2	20	18	-
	3	-	34	-
	5	32	-	-
LPS [µM]	10	25	15	1
	20	47	24	16
	50	52	18	9
	100	73	15	9
*E. coli* [µL]	2	3	-	-
	4	0	-	-
	8	2	-	-
	12	3	-	-

**Table 3:** Antibacterial activity of the peptides and antibiotics expressed as their MIC.

Peptide	MIC [µM]	
	*E. coli* K12	*E. coli* BL21(DE3)
anoplin	32	32
W-MreB_1–9_	128	256
(KFF)_3_K	32	32
ampicillin	8	4
tetracycline	8	16

## Supplementary Materials

Supplementary materials can be found at https://www.mdpi.com/1422-0067/21/24/9672/s1.

## Abbreviations

AMP	Antimicrobial peptide
CD	Circular dichroism
CMC	Critical micelle concentration
CPP	Cell-penetrating peptide
DPC	Dodecylphosphocholine
HT	High tension
LB	Lysogeny broth
LPS	Lipopolysacharide
LUVs	Large unilamellar liposomes
MIC	Minimum inhibitory concentration
MMW NRMSD	Measured molecular weight Normalized root mean square deviation
MS	Mass spectrometry
POPC	2-Oleoyl-1-palmitoyl-*sn*-glycero-3-phosphocholine
POPE	2-Oleoyl-1-palmitoyl-*sn*-glycero-3-phosphoethanolamine
POPG	2-Oleoyl-1-palmitoyl-*sn*-glycero-3-phospho-rac-(1-glycerol)
SDS	Sodium dodecyl sulfate
SPPS	Solid-phase peptide synthesis
SUVs	Small unilamellar vesicles
RP-HPLC	Reverse phase-high-performance liquid chromatography
RT	Retention time
TMW	Theoretical molecular weight
