# Supplementary material for: Conformational Changes of Anoplin, W-MreB1–9, and (KFF)3K Peptides near the Membranes
Source: Int J Mol Sci. 2020 Dec 18;21(24):9672. doi: 10.3390/ijms21249672 (PMC7766051; doi:10.3390/ijms21249672)
Supplement: Supplementary file 1 [file ijms-21-09672-s001.pdf]

International Journal of *Molecular Sciences*

MDPI

Article

Conformational Changes of Anoplin, W-MreB$_{1-9}$, and (KFF)$_3$K Peptides near the Membranes

Monika Wojciechowska [1,*], Joanna Miszkiewicz [1,2] and Joanna Trylska [1,*]

1 Centre of New Technologies, University of Warsaw, Banacha 2c, 02-097, Warsaw, Poland;
2 College of Inter-Faculty Individual Studies in Mathematics and Natural Sciences, University of Warsaw, Banacha 2c, 02-097, Warsaw, Poland
* Correspondence: m.wojciechowska@cent.uw.edu.pl (M.W.); joanna@cent.uw.edu.pl (J.T.)

Supplementary Materials

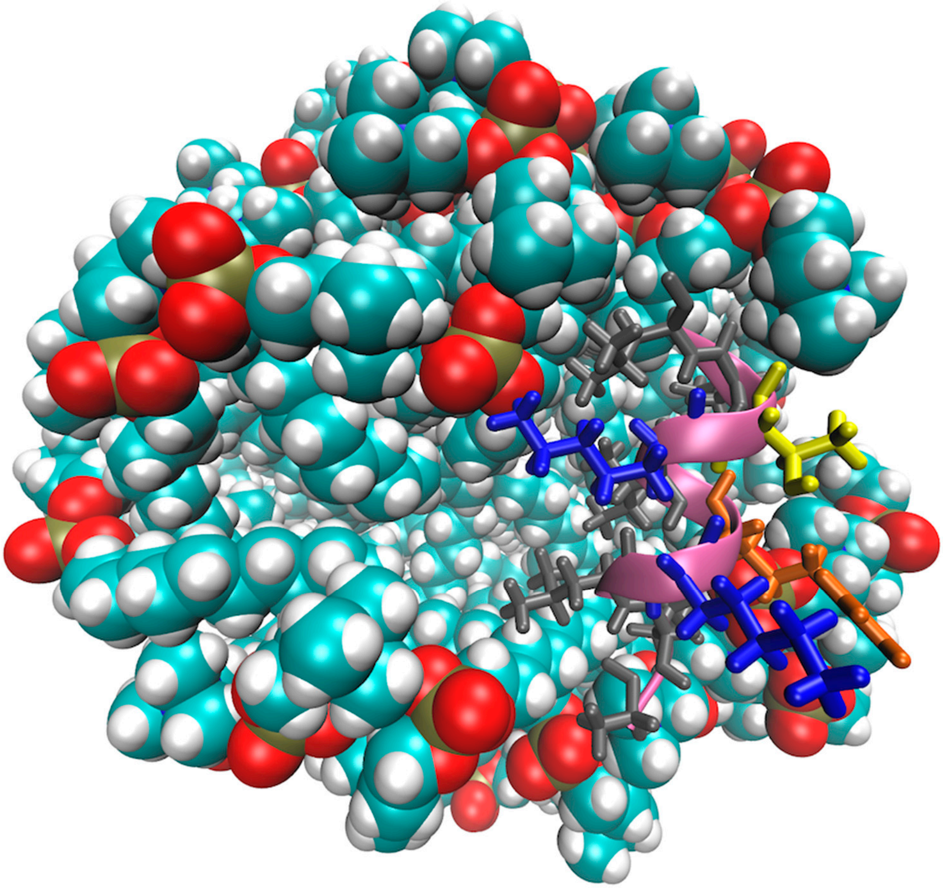

Figure S1. The model of anoplin bound to a DPC micelle visualized based on the solution NMR structure with PDB ID 2MJQ [26]. Anoplin forms a helix marked as pink ribbon, with the Lys side-chains shown in blue, Arg in orange, Thr in yellow, and Leu, Ile, and Gly in grey. The central Lys amine hydrogen bonds with the phosphate oxygen of the micelle. The figure was prepared with VMD (https://www.ks.uiuc.edu/Research/vmd/).

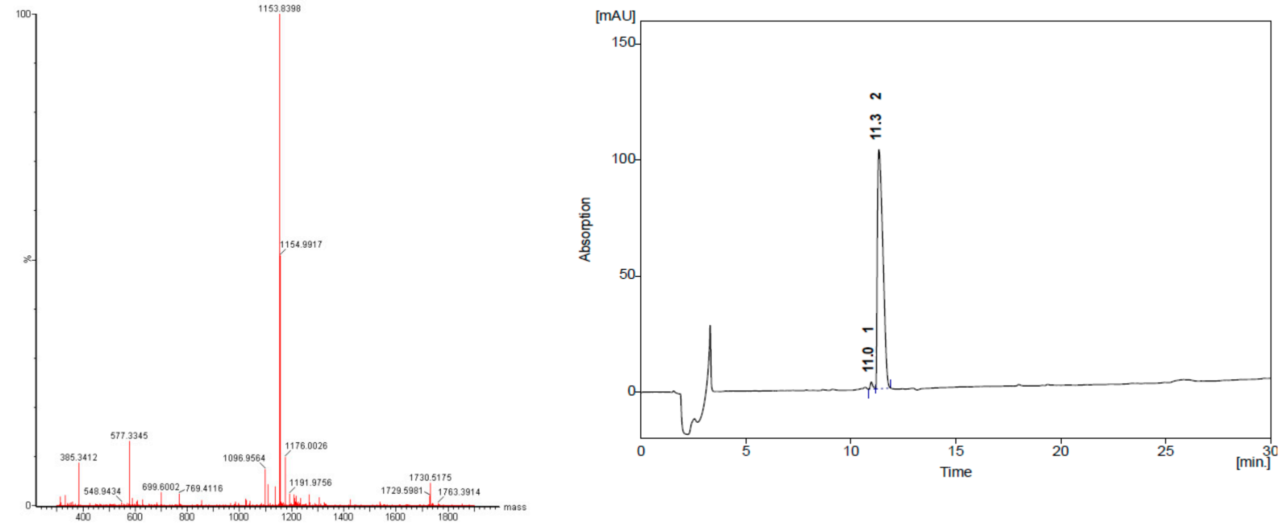

Figure S2. Mass spectrum and RP-HPLC chromatogram of anopiln after purification. For the HPLC method see section "Materials and Methods", subsection 4.1 in the main text "*Peptide synthesis and purification*".

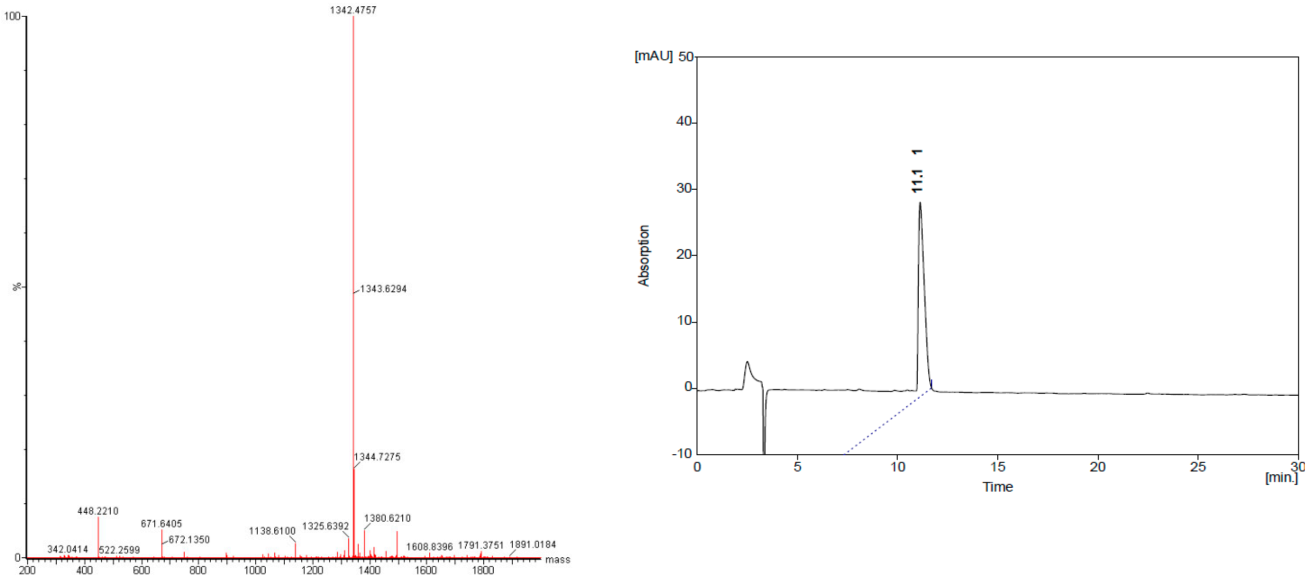

Figure S3. Mass spectrum and RP-HPLC chromatogram of W-MreB$_{1-9}$ after purification. For the HPLC method see section "Materials and Methods", subsection 4.1 in the main text "*Peptide synthesis and purification*".

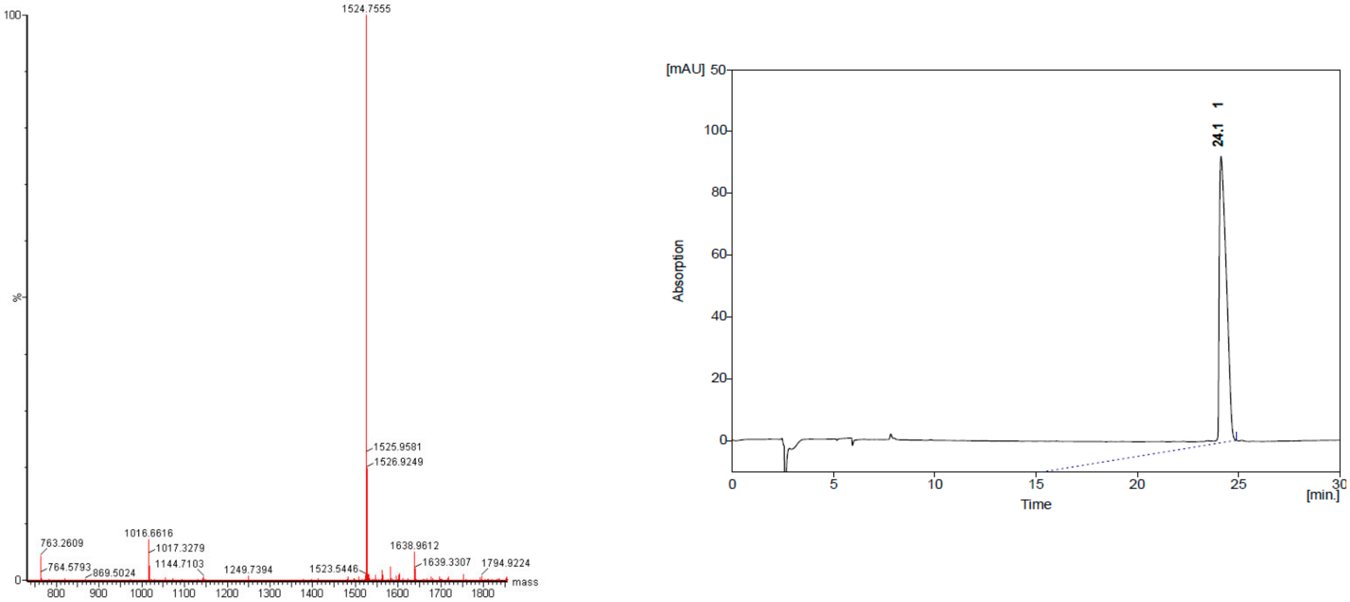

Figure S4. Mass spectrum and RP-HPLC chromatogram of (KFF)3K after purification. For the HPLC method see section "Materials and Methods", subsection 4.1 in the main text "*Peptide synthesis and purification*".

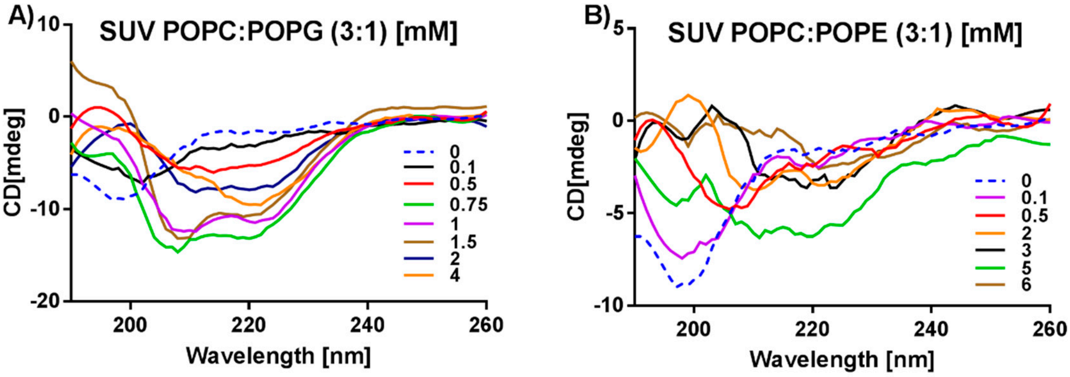

Figure S5. CD spectra of anoplin in the presence of **A)** POPC:POPG (3:1) and **B)** POPC:POPE (3:1) SUVs with the legends showing the lipids concentrations in mM.

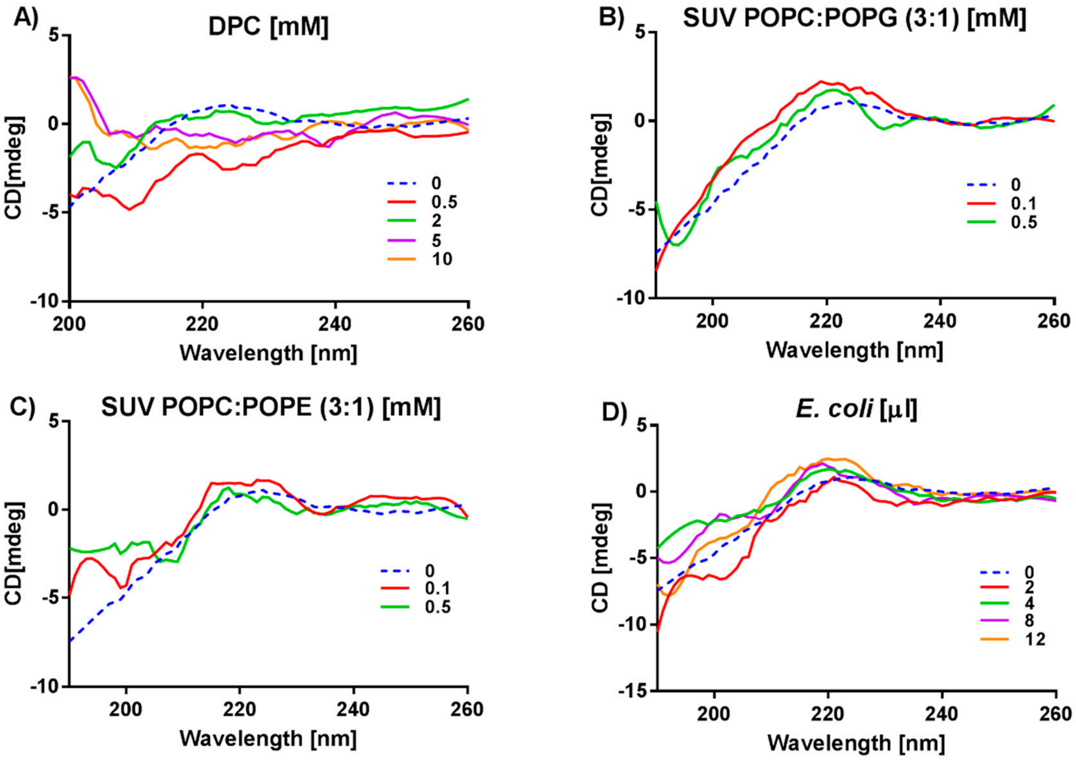

Figure S6. CD spectra of (KFF)3K in the presence of **A)** DPC micelles, **B)** POPC:POPG (3:1) SUVs, **C)** POPC:POPE (3:1) SUVs, and **D)** *E. coli* BL21(DE3) cells. The legends show the membrane concentrations.

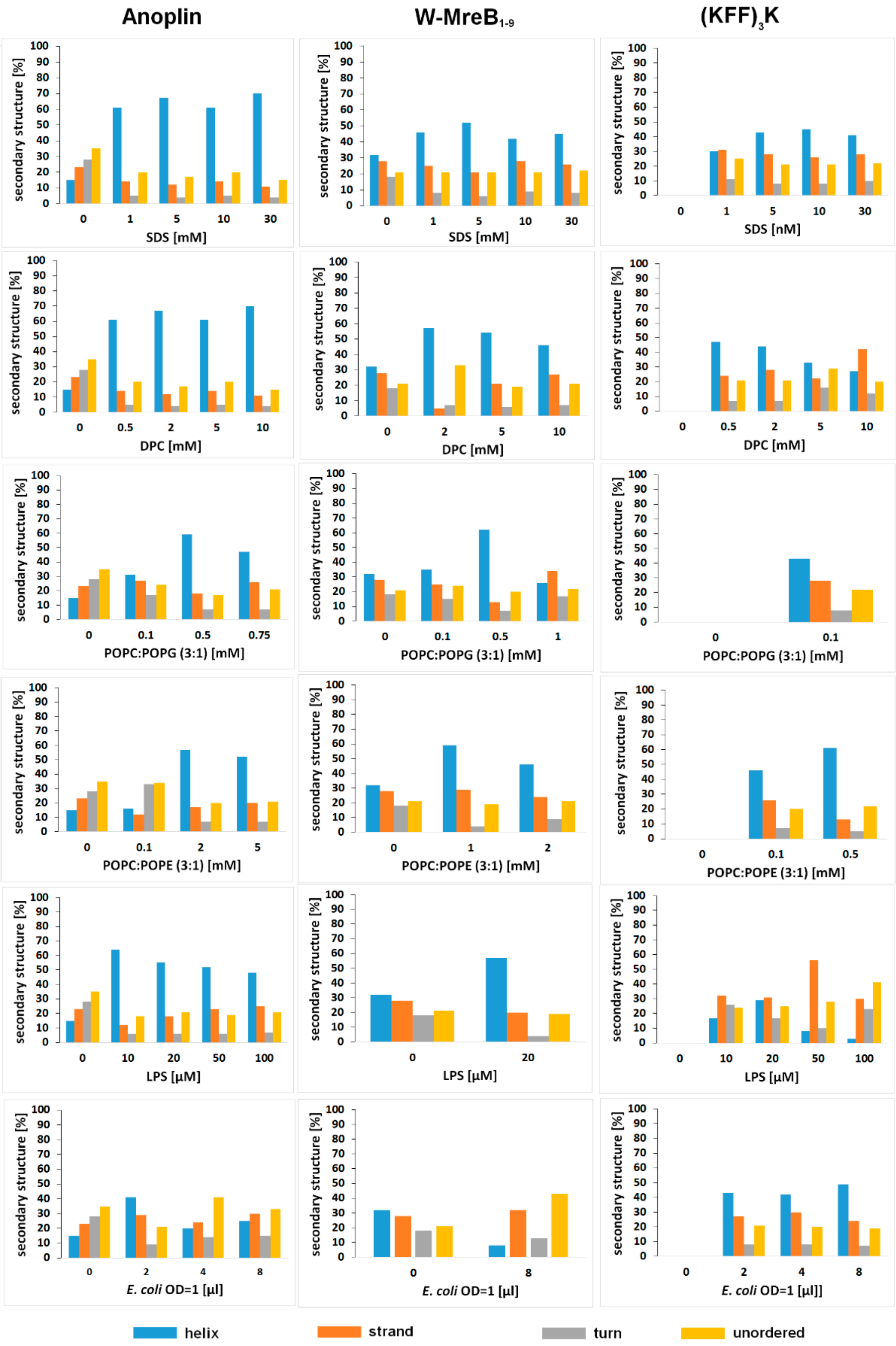

Figure S7. Secondary structure contents (in percent) in the peptide structures determined from the DichroWeb server using the CDSSTR algorithm and reference data set 4 [47,48]. The percentages of helices and strands are the sum of the contributions of the regular and distorted α-helices or the regular and distorted β-sheets, respectively. The percentages of turns and unordered structures were taken directly from the output data.

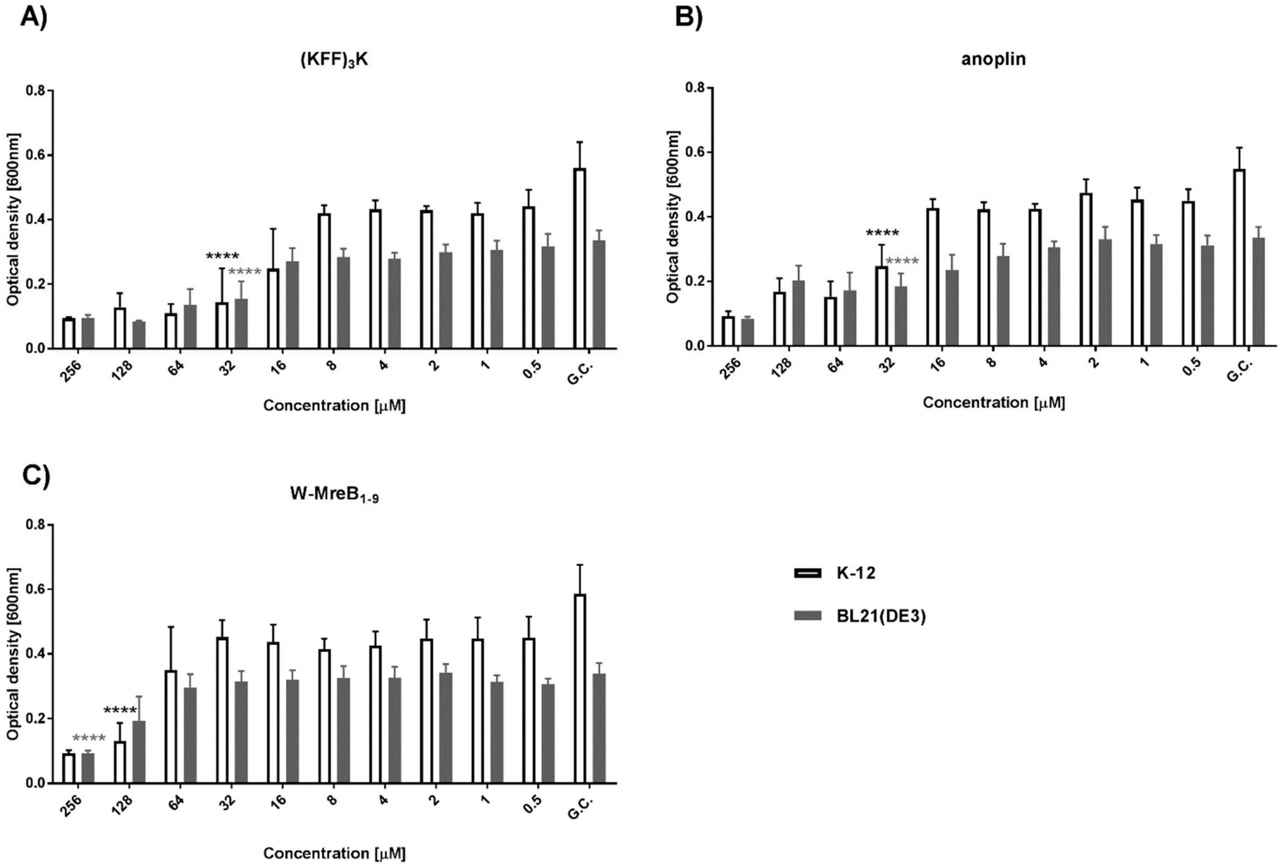

Figure S8. Optical density (OD) as a measure of *E. coli* K12 and BL21(DE3) growth shown after 20 h incubation with various concentrations of $(KFF)_3K$, anoplin, and W-$MreB_{1-9}$ peptides. Error bars represent the standard error of the mean. G.C. stands for growth control. The **** mark the MIC.

A) For the K12 strain, the OD differences between the G.C. and samples treated with different $(KFF)_3K$ concentrations were statistically highly significant ($P < 0.001$ for 0.5 µM and $P < 0.0001$ for all other $(KFF)_3K$ concentrations). For the BL21 strain, the OD differences between the G.C. and 0.5 µM – 8 µM $(KFF)_3K$ were not significant ($P > 0.05$), between G.C. and 16 µM $(KFF)_3K$ were marginally significant ($P < 0.05$), and between G.C. and 32 – 256 µM were highly significant ($P < 0.0001$).

B) For K12, the OD differences between the G.C. and samples treated with anoplin were statistically highly significant ($P < 0.0001$) except for anoplin concentration of 2 µM ($P < 0.05$). For BL21, the OD differences between the G.C. and 0.5 µM – 8 µM anoplin samples were not significant, and between G.C. and anoplin concentrations ≥ 16 µM were highly significant ($P < 0.0001$).

C) For K12, the OD differences between the G.C. and samples treated with W-$MreB_{1-9}$ were statistically highly significant ($P < 0.0001$). For BL21, the OD differences between the G.C. and $MreB_{1-9}$ concentrations up to 64 µM were insignificant ($P > 0.05$), and for higher $MreB_{1-9}$ concentrations they were highly statistically significant ($P < 0.0001$).

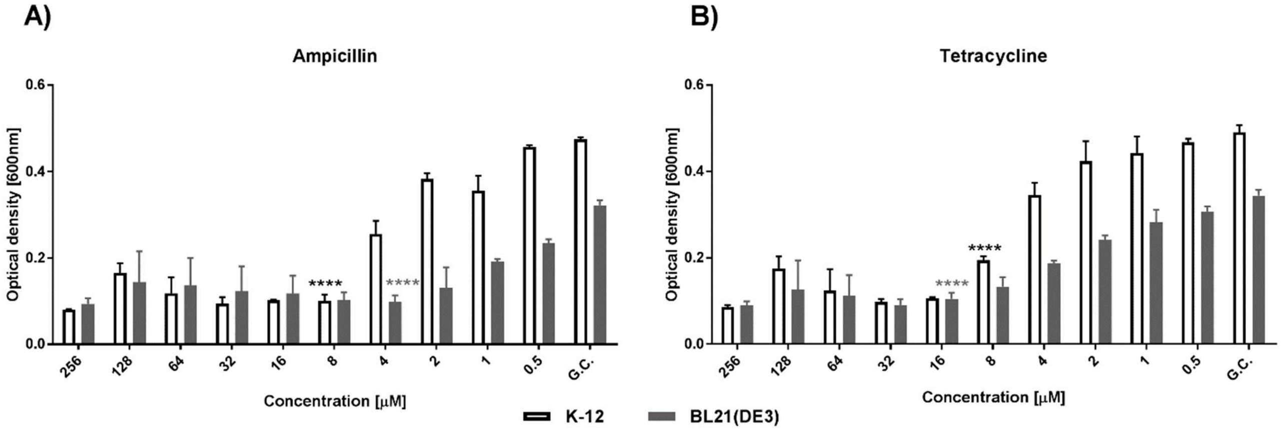

Figure S9. Optical density (OD) as a measure of *E. coli* K12 and BL21(DE3) growth shown after 20 h incubation with various concentrations of ampicillin or tetracycline. Error bars represent the standard error of the mean. G.C. stands for growth control. The **** mark the MIC concentrations.

A) For the K12 strain, the OD differences between the G.C. and samples with ampicillin concentrations starting from 4 μM were highly statistically significant ($P < 0.0001$). For BL21, the differences between the G.C. and samples for ampicillin concentrations ≥ 2 μM were highly significant ($P < 0.0001$).

B) For K12, the differences between the G.C. and samples treated with tetracycline concentrations up to 2 μM were statistically insignificant ($P > 0.05$) and for ≥ 4 μM were highly significant ($P < 0.0001$). For BL21, the differences between the G.C. and tetracycline concentrations up to 1 μM were statistically insignificant ($P > 0.05$), and for higher tetracycline concentrations they were highly significant.

Table S1. Fractions of secondary structures in anoplin in different surroundings calculated from DichroWeb [47,48]. The experimental CD spectra are shown in Figure 2 in the main text.

Anoplin		Calculated secondary structure fractions							NRMSD[1]
		Method / Reference set.	α-helix regular	α-helix distorted	β-sheet regular	β-sheet distorted	Turns	Unordered	
buffer		CONTINLL/4	0.000	0.360	0.000	0.077	0.303	0.260	0.176
		CONTINLL/7	0.000	0.208	0.000	0.001	0.000	0.790	0.098
		CONTINLL/SP180	0.000	0.162	0.000	0.107	0.197	0.535	0.206
		CDSSTR/4	0.04	0.11	0.10	0.13	0.28	0.35	0.001
		CDSSTR/7	0.35	0.10	0.14	0.10	0.10	0.22	0.001
		CDSSTR/SP180	0.42	0.13	0.06	0.08	0.11	0.22	0.001
SDS [mM]	1	CONTINLL/4	0.523	0.404	0.000	0.073	0.000	0.000	0.142
		CONTINLL/7	0.596	0.404	0.000	0.000	0.000	0.000	0.142
		CONTINLL/SP180	0.362	0.352	0.000	0.000	0.082	0.204	0.162
		CDSSTR/4	0.48	0.13	0.07	0.07	0.05	0.20	0.001
		CDSSTR/7	0.46	0.14	0.07	0.07	0.06	0.20	0.001
		CDSSTR/SP180	0.43	0.11	0.12	0.09	0.08	0.18	0.001
	5	CONTINLL/4	0.490	0.510	0.000	0.000	0.000	0.000	0.107
		CONTINLL/7	0.591	0.409	0.000	0.000	0.000	0.000	0.107
		CONTINLL/SP180	0.473	0.527	0.000	0.000	0.000	0.000	0.114
		CDSSTR/4	0.52	0.15	0.06	0.06	0.04	0.17	0.000
		CDSSTR/7	0.50	0.16	0.06	0.06	0.05	0.18	0.000
		CDSSTR/SP180	0.45	0.11	0.13	0.09	0.08	0.15	0.000
	10	CONTINLL/4	0.412	0.588	0.000	0.000	0.000	0.000	0.136

		CONTINLL/7	0.537	0.463	0.000	0.000	0.000	0.000	0.136
		CONTINLL/SP180	0.000	0.671	0.000	0.000	0.152	0.178	0.146
		CDSSTR/4	0.48	0.13	0.07	0.07	0.05	0.20	0.000
		CDSSTR/7	0.47	0.13	0.09	0.08	0.05	0.20	0.000
		CDSSTR/SP180	0.41	0.10	0.14	0.10	0.08	0.19	0.000
	30	CONTINLL/4	0.509	0.491	0.000	0.000	0.000	0.000	0.103
		CONTINLL/7	0.604	0.396	0.000	0.000	0.000	0.000	0.103
		CONTINLL/SP180	-	-	-	-	-	-	-
		CDSSTR/4	0.54	0.16	0.04	0.07	0.04	0.15	0.000
		CDSSTR/7	0.52	0.17	0.03	0.06	0.04	0.17	0.000
		CDSSTR/SP180	0.47	0.13	0.13	0.08	0.08	0.12	0.000
DPC [mM]	0.5	CONTINLL/4	0.062	0.374	0.000	0.131	0.433	0.000	0.376
		CONTINLL/7	0.098	0.403	0.000	0.113	0.387	0.000	0.376
		CONTINLL/SP180	0.079	0.168	0.000	0.090	0.181	0.481	0.427
		CDSSTR/4	0.36	0.09	0.14	0.11	0.09	0.21	0.002
		CDSSTR/7	0.44	0.16	0.10	0.07	0.06	0.15	0.002
		CDSSTR/SP180	0.45	0.14	0.07	0.05	0.13	0.15	0.004
	2	CONTINLL/4	0.628	0.372	0.000	0.000	0.000	0.000	0.099
		CONTINLL/7	0.694	0.306	0.000	0.000	0.000	0.000	0.099
		CONTINLL/SP180	0.583	0.417	0.000	0.000	0.000	0.000	0.112
		CDSSTR/4	0.55	0.16	0.05	0.06	0.04	0.13	0.001
		CDSSTR/7	0.51	0.17	0.05	0.07	0.06	0.15	0.000
		CDSSTR/SP180	0.54	0.12	0.08	0.06	0.05	0.14	0.000
	5	CONTINLL/4	0.619	0.381	0.000	0.000	0.000	0.000	0.057
		CONTINLL/7	0.629	0.371	0.000	0.000	0.000	0.000	0.057
		CONTINLL/SP180	0.422	0.417	0.000	0.000	0.042	0.118	0.079
		CDSSTR/4	0.52	0.12	0.06	0.08	0.07	0.16	0.000
		CDSSTR/7	0.51	0.15	0.05	0.07	0.06	0.17	0.000
		CDSSTR/SP180	0.47	0.16	0.12	0.08	0.07	0.10	0.000
	10	CONTINLL/4	0.557	0.443	0.000	0.000	0.000	0.000	0.073
		CONTINLL/7	0.615	0.385	0.000	0.000	0.000	0.000	0.073
		CONTINLL/SP180	0.401	0.438	0.000	0.000	0.041	0.119	0.091
		CDSSTR/4	0.51	0.13	0.05	0.08	0.05	0.18	0.000
		CDSSTR/7	0.50	0.16	0.04	0.06	0.05	0.19	0.000
		CDSSTR/SP180	0.45	0.12	0.13	0.08	0.08	0.14	0.000
POPC:POPG (3:1) [mM]	0.1	CONTINLL/4	0.000	0.365	0.000	0.057	0.386	0.192	0.236
		CONTINLL/7	0.000	0.219	0.000	0.000	0.000	0.781	0.121
		CONTINLL/SP180	0.000	0.321	0.000	0.040	0.169	0.470	0.196
		CDSSTR/4	0.23	0.09	0.16	0.11	0.17	0.24	0.001
		CDSSTR/7	0.44	0.17	0.05	0.06	0.07	0.20	0.001
		CDSSTR/SP180	0.43	0.13	0.06	0.08	0.09	0.20	0.001
	0.5	CONTINLL/4	0.339	0.324	0.000	0.037	0.190	0.111	0.097
		CONTINLL/7	0.348	0.331	0.000	0.079	0.242	0.000	0.097

		CONTINLL/SP180	0.208	0.304	0.000	0.025	0.137	0.326	0.288
		CDSSTR/4	0.47	0.12	0.09	0.09	0.07	0.17	0.001
		CDSSTR/7	0.49	0.15	0.07	0.08	0.05	0.17	0.001
		CDSSTR/SP180	0.42	0.15	0.07	0.08	0.11	0.17	0.001
	0.75	CONTINLL/4	0.000	0.000	0.000	0.000	1.000	0.000	0.151
		CONTINLL/7	0.488	0.512	0.000	0.000	0.000	0.000	0.151
		CONTINLL/SP180	0.000	1.000	0.000	0.000	0.000	0.000	0.322
		CDSSTR/4	0.39	0.08	0.15	0.11	0.07	0.21	0.000
		CDSSTR/7	0.40	0.08	0.14	0.11	0.07	0.21	0.000
		CDSSTR/SP180	0.37	0.07	0.18	0.12	0.07	0.21	0.000
POPC:POPE (3:1) [mM]	0.1	CONTINLL/4	0.000	1.000	0.000	0.000	0.000	0.000	0.205
		CONTINLL/7	0.000	0.272	0.000	0.000	0.000	0.728	0.106
		CONTINLL/SP180	0.000	0.000	0.000	0.000	1.000	0.000	0.792
		CDSSTR/4	0.15	0.01	0.03	0.09	0.33	0.34	0.001
		CDSSTR/7	0.40	0.15	0.07	0.07	0.06	0.25	0.000
		CDSSTR/SP180	0.41	0.11	0.10	0.08	0.08	0.23	0.000
	2	CONTINLL/4	0.676	0.324	0.000	0.000	0.000	0.000	0.270
		CONTINLL/7	1.000	0.000	0.000	0.000	0.000	0.000	0.270
		CONTINLL/SP180	0.677	0.323	0.000	0.000	0.000	0.000	0.357
		CDSSTR/4	0.47	0.10	0.09	0.08	0.07	0.20	0.001
		CDSSTR/7	0.45	0.10	0.10	0.08	0.07	0.21	0.001
		CDSSTR/SP180	0.51	0.11	0.05	0.08	0.08	0.17	0.001
	5	CONTINLL/4	0.314	0.497	0.000	0.055	0.134	0.000	0.424
		CONTINLL/7	0.242	0.525	0.000	0.117	0.116	0.000	0.199
		CONTINLL/SP180	0.000	0.381	0.000	0.000	0.269	0.349	0.434
		CDSSTR/4	0.43	0.09	0.10	0.10	0.07	0.21	0.000
		CDSSTR/7	0.42	0.09	0.10	0.10	0.07	0.22	0.000
		CDSSTR/SP180	0.37	0.08	0.17	0.12	0.07	0.22	0.000
LPS [µM]	10	CONTINLL/4	0.284	0.539	0.000	0.000	0.167	0.011	0.301
		CONTINLL/7	0.258	0.412	0.000	0.000	0.000	0.330	0.153
		CONTINLL/SP180	0.000	0.394	0.000	0.046	0.161	0.399	0.324
		CDSSTR/4	0.51	0.13	0.03	0.09	0.06	0.18	0.000
		CDSSTR/7	0.45	0.16	0.06	0.07	0.07	0.21	0.000
		CDSSTR/SP180	0.39	0.13	0.12	0.09	0.08	0.21	0.000
	20	CONTINLL/4	0.535	0.465	0.000	0.000	0.000	0.000	0.116
		CONTINLL/7	0.601	0.399	0.000	0.000	0.000	0.000	0.116
		CONTINLL/SP180	0.349	0.651	0.000	0.000	0.000	0.000	0.147
		CDSSTR/4	0.45	0.11	0.09	0.09	0.06	0.21	0.000
		CDSSTR/7	0.43	0.10	0.10	0.09	0.06	0.22	0.000
		CDSSTR/SP180	0.40	0.08	0.16	0.11	0.07	0.19	0.000
	50	CONTINLL/4	1.000	0.000	0.000	0.000	0.000	0.000	0.058
		CONTINLL/7	1.000	0.000	0.000	0.000	0.000	0.000	0.058
		CONTINLL/SP180	-	-	-	-	-	-	-

		CDSSTR/4	0.43	0.09	0.12	0.11	0.06	0.19	0.000
		CDSSTR/7	0.40	0.08	0.15	0.11	0.06	0.21	0.000
		CDSSTR/SP180	0.41	0.09	0.16	0.11	0.07	0.18	0.000
	100	CONTINLL/4	0.522	0.478	0.000	0.000	0.000	0.000	0.160
		CONTINLL/7	0.548	0.452	0.000	0.000	0.000	0.000	0.160
		CONTINLL/SP180	0.000	1.000	0.000	0.000	0.000	0.000	0.098
		CDSSTR/4	0.40	0.08	0.14	0.11	0.07	0.21	0.000
		CDSSTR/7	0.39	0.08	0.15	0.11	0.07	0.21	0.000
		CDSSTR/SP180	0.37	0.07	0.18	0.12	0.07	0.21	0.000
E. coli OD=1 [µl]	2	CONTINLL/4	0.000	0.621	0.000	0.044	0.335	0.000	0.250
		CONTINLL/7	0.000	0.000	0.000	0.000	0.000	1.000	0.241
		CONTINLL/SP180	0.000	0.247	0.000	0.043	0.140	0.571	0.327
		CDSSTR/4	0.34	0.07	0.17	0.12	0.09	0.21	0.000
		CDSSTR/7	0.41	0.12	0.07	0.08	0.03	0.30	0.001
		CDSSTR/SP180	0.41	0.12	0.07	0.09	0.10	0.22	0.001
	4	CONTINLL/4	0.000	0.370	0.000	0.192	0.438	0.000	0.174
		CONTINLL/7	0.000	0.170	0.000	0.050	0.000	0.780	0.138
		CONTINLL/SP180	0.000	0.157	0.000	0.063	0.165	0.614	0.273
		CDSSTR/4	0.01	0.19	0.10	0.14	0.14	0.41	0.003
		CDSSTR/7	0.33	0.06	0.15	0.10	0.07	0.29	0.000
		CDSSTR/SP180	0.43	0.14	0.05	0.08	0.12	0.20	0.001
	8	CONTINLL/4	0.000	0.412	0.000	0.000	0.000	0.588	0.226
		CONTINLL/7	0.000	0.227	0.000	0.000	0.000	0.773	0.194
		CONTINLL/SP180	-	-	-	-	-	-	-
		CDSSTR/4	0.11	0.14	0.14	0.16	0.15	0.33	0.001
		CDSSTR/7	0.15	0.05	0.11	0.11	0.17	0.41	0.001
		CDSSTR/SP180	-	-	-	-	-	-	-
	12	CONTINLL/4	0.000	0.369	0.000	0.019	0.290	0.322	0.107
		CONTINLL/7	0.000	0.204	0.000	0.000	0.000	0.796	0.114
		CONTINLL/SP180	0.000	0.218	0.000	0.070	0.146	0.566	0.198
		CDSSTR/4	0.02	0.20	0.13	0.07	0.27	0.31	0.002
		CDSSTR/7	0.34	0.09	0.14	0.10	0.09	0.25	0.000
		CDSSTR/SP180	0.40	0.11	0.08	0.08	0.11	0.21	0.001

[1] normalized root mean square deviation. The NRMSD value less than 0.1 suggests a good fit of anoplin CD spectrum to the reference set, and NRSMD above 0.5 suggests that the peptide CD spectrum does not fit well to the CD spectra of the reference set. But low NRMSD do not ensure that the analysis is accurate [47].

Table S2. Fractions of secondary structures in W-MreB$_{1-9}$ in different surroundings calculated from DichroWeb [47,48]. The experimental CD spectra are shown in Figure 3 in the main text.

W-MeB$_{1-9}$	Calculated secondary structure fractions							NRMSD[1]
	Method / Reference set.	α-helix regular	α-helix distorted	β-sheet regular	β-sheet distorted	Turns	Unordered	

buffer		CONTINLL/4	0.178	0.425	0.000	0.000	0.220	0.177	0.261
		CONTINLL/7	0.456	0.544	0.000	0.000	0.000	0.000	0.148
		CONTINLL/SP180	-	-	-	-	-	-	-
		CDSSTR/4	0.22	0.10	0.18	0.10	0.18	0.21	0.001
		CDSSTR/7	0.41	0.17	0.08	0.07	0.05	0.20	0.001
		CDSSTR/SP180	0.43	0.14	0.06	0.07	0.10	0.20	0.000
SDS [mM]	1	CONTINLL/4	0.000	0.620	0.000	0.084	0.296	0.000	0.476
		CONTINLL/7	0.000	0.404	0.000	0.000	0.000	0.596	0.293
		CONTINLL/SP180	0.000	0.326	0.000	0.000	0.330	0.344	0.339
		CDSSTR/4	0.38	0.08	0.15	0.10	0.08	0.21	0.000
		CDSSTR/7	0.39	0.13	0.09	0.08	0.08	0.23	0.001
		CDSSTR/SP180	0.43	0.12	0.07	0.07	0.11	0.21	0.001
	5	CONTINLL/4	0.000	1.000	0.000	0.000	0.000	0.000	0.530
		CONTINLL/7	0.000	1.000	0.000	0.000	0.000	0.000	0.245
		CONTINLL/SP180	0.39	0.08	0.14	0.11	0.09	0.21	0.000
		CDSSTR/4	0.41	0.11	0.12	0.09	0.06	0.21	0.000
		CDSSTR/7	0.39	0.13	0.11	0.09	0.06	0.21	0.000
		CDSSTR/SP180	0.39	0.08	0.14	0.11	0.09	0.21	0.000
	10	CONTINLL/4	0.000	0.000	0.000	0.000	1.000	0.000	0.467
		CONTINLL/7	0.000	0.616	0.000	0.000	0.384	0.000	0.258
		CONTINLL/SP180	0.000	0.489	0.000	0.029	0.085	0.396	0.584
		CDSSTR/4	0.35	0.07	0.17	0.11	0.09	0.21	0.000
		CDSSTR/7	0.38	0.14	0.12	0.09	0.07	0.20	0.001
		CDSSTR/SP180	0.40	0.10	0.12	0.10	0.09	0.21	0.000
	30	CONTINLL/4	0.017	0.499	0.000	0.000	0.277	0.207	0.398
		CONTINLL/7	0.036	0.411	0.000	0.000	0.099	0.455	0.159
		CONTINLL/SP180	0.000	0.316	0.000	0.098	0.143	0.443	0.474
		CDSSTR/4	0.37	0.08	0.15	0.11	0.08	0.22	0.000
		CDSSTR/7	0.40	0.16	0.05	0.05	0.07	0.27	0.001
		CDSSTR/SP180	0.42	0.13	0.08	0.07	0.09	0.22	0.001
DPC [mM]	0.5	CONTINLL/4	0.000	0.271	0.000	0.084	0.191	0.454	0.187
		CONTINLL/7	0.000	0.136	0.000	0.028	0.000	0.836	0.054
		CONTINLL/SP180	0.000	0.105	0.000	0.098	0.118	0.679	0.253
		CDSSTR/4	-	-	-	-	-	-	-
		CDSSTR/7	-	-	-	-	-	-	-
		CDSSTR/SP180	0.34	0.06	0.17	0.12	0.07	0.25	0.000
	2	CONTINLL/4	0.229	0.536	0.000	0.005	0.230	0.000	0.250
		CONTINLL/7	0.386	0.513	0.000	0.101	0.000	0.000	0.250
		CONTINLL/SP180	0.000	0.001	0.733	0.266	0.000	0.000	0.770
		CDSSTR/4	0.51	0.06	0	0.06	0.07	0.33	0.001
		CDSSTR/7	0.39	0.09	0.14	0.10	0.07	0.20	0.000
		CDSSTR/SP180	0.40	0.09	0.15	0.10	0.08	0.19	0.000
	5	CONTINLL/4	0.537	0.463	0.000	0.000	0.000	0.000	0.158

		CONTINLL/7	0.579	0.421	0.000	0.000	0.000	0.000	0.158
		CONTINLL/SP180	-	-	-	-	-	-	-
		CDSSTR/4	0.44	0.10	0.12	0.09	0.06	0.19	0.000
		CDSSTR/7	0.44	0.12	0.10	0.08	0.07	0.19	0.000
		CDSSTR/SP180	0.46	0.09	0.12	0.09	0.07	0.18	0.000
	10	CONTINLL/4	0.408	0.592	0.000	0.000	0.000	0.000	0.121
		CONTINLL/7	1.000	0.000	0.000	0.000	0.000	0.000	0.121
		CONTINLL/SP180	0.000	1.000	0.000	0.000	0.000	0.000	0.170
		CDSSTR/4	0.39	0.07	0.16	0.11	0.07	0.21	0.000
		CDSSTR/7	0.39	0.09	0.15	0.11	0.07	0.20	0.000
		CDSSTR/SP180	0.39	0.08	0.17	0.11	0.07	0.19	0.000
POPC:POPG (3:1) [mM]	0.1	CONTINLL/4	0.093	0.246	0.019	0.000	0.211	0.432	0.269
		CONTINLL/7	0.000	0.378	0.622	0.000	0.000	0.000	0.136
		CONTINLL/SP180	0.000	0.324	0.000	0.107	0.069	0.501	0.374
		CDSSTR/4	0.26	0.09	0.16	0.09	0.15	0.24	0.001
		CDSSTR/7	0.40	0.15	0.10	0.08	0.08	0.19	0.001
		CDSSTR/SP180	0.41	0.13	0.08	0.07	0.09	0.22	0.001
	0.5	CONTINLL/4	0.000	0.000	0.000	0.000	0.000	1.000	0.208
		CONTINLL/7	0.550	0.264	0.000	0.061	0.125	0.000	0.208
		CONTINLL/SP180	0.284	0.359	0.000	0.022	0.110	0.224	0.171
		CDSSTR/4	0.51	0.11	0.03	0.10	0.07	0.20	0.000
		CDSSTR/7	0.43	0.10	0.10	0.10	0.06	0.21	0.000
		CDSSTR/SP180	0.39	0.11	0.17	0.11	0.08	0.15	0.000
	1	CONTINLL/4	0.144	0.269	0.000	0.000	0.116	0.471	0.385
		CONTINLL/7	0.202	0.298	0.000	0.000	0.156	0.344	0.165
		CONTINLL/SP180	0.676	0.324	0.000	0.000	0.000	0.000	0.357
		CDSSTR/4	0.24	0.02	0.20	0.14	0.17	0.22	0.000
		CDSSTR/7	0.42	0.13	0.17	0.09	0.07	0.14	0.000
		CDSSTR/SP180	0.38	0.08	0.16	0.11	0.08	0.20	0.000
POPC:POPE (3:1) [mM]	1	CONTINLL/4	0.579	0.233	0.000	0.000	0.000	0.188	0.268
		CONTINLL/7	0.000	0.000	0.000	0.000	1.000	0.000	0.225
		CONTINLL/SP180	0.277	0.319	0.000	0.000	0.066	0.337	0.319
		CDSSTR/4	0.47	0.12	0.11	0.08	0.04	0.19	0.001
		CDSSTR/7	0.45	0.13	0.10	0.08	0.05	0.17	0.001
		CDSSTR/SP180	0.47	0.11	0.10	0.07	0.11	0.15	0.000
	2	CONTINLL/4	0.236	0.443	0.000	0.052	0.182	0.087	0.511
		CONTINLL/7	0.342	0.507	0.000	0.151	0.000	0.000	0.177
		CONTINLL/SP180	0.043	0.285	0.000	0.031	0.223	0.418	0.475
		CDSSTR/4	0.38	0.08	0.13	0.11	0.09	0.21	0.000
		CDSSTR/7	0.43	0.12	0.08	0.08	0.08	0.21	0.001
		CDSSTR/SP180	0.41	0.10	0.11	0.09	0.09	0.19	0.001
	3	CONTINLL/4	0.000	0.000	0.000	0.000	1.000	0.000	0.205
		CONTINLL/7	0.000	0.000	1.000	0.000	0.000	0.000	0.205

		CONTINLL/SP180	0.000	0.779	0.000	0.000	0.221	0.000	0.449
		CDSSTR/4	-	-	-	-	-	-	-
		CDSSTR/7	0.40	0.09	0.15	0.10	0.06	0.21	0.000
		CDSSTR/SP180	0.38	0.08	0.17	0.11	0.08	0.20	0.000
LPS [µM]	10	CONTINLL/4	0.000	0.000	0.000	0.360	0.640	0.000	0.540
		CONTINLL/7	0.032	0.133	0.043	0.096	0.322	0.374	0.194
		CONTINLL/SP180	-	-	-	-	-	-	-
		CDSSTR/4	-	-	-	-	-	-	-
		CDSSTR/7	0.43	0.09	0.09	0.11	0.08	0.18	0.000
		CDSSTR/SP180	0.39	0.09	0.14	0.10	0.08	0.21	0.000
	20	CONTINLL/4	0.000	0.629	0.000	0.371	0.000	0.000	0.278
		CONTINLL/7	0.210	0.684	0.000	0.106	0.000	0.000	0.226
		CONTINLL/SP180	0.000	0.556	0.000	0.119	0.000	0.326	0.377
		CDSSTR/4	0.46	0.11	0.12	0.08	0.04	0.19	0.001
		CDSSTR/7	0.41	0.11	0.10	0.08	0.07	0.23	0.000
		CDSSTR/SP180	0.44	0.09	0.12	0.09	0.07	0.20	0.000
	50	CONTINLL/4	0.000	0.000	0.000	0.000	1.000	0.000	0.313
		CONTINLL/7	1.000	0.000	0.000	0.000	0.000	0.000	0.288
		CONTINLL/SP180	0.637	0.292	0.000	0.071	0.000	0.000	0.406
		CDSSTR/4	-	-	-	-	-	-	-
		CDSSTR/7	0.35	0.06	0.19	0.12	0.08	0.21	0.000
		CDSSTR/SP180	0.44	0.09	0.14	0.08	0.08	0.17	0.001
	100	CONTINLL/4	0.020	0.000	0.155	0.194	0.154	0.477	0.271
		CONTINLL/7	0.000	0.000	0.297	0.284	0.419	0.000	0.271
		CONTINLL/SP180	0.003	0.000	0.773	0.194	0.000	0.030	0.223
		CDSSTR/4	-	-	-	-	-	-	-
		CDSSTR/7	-	-	-	-	-	-	-
		CDSSTR/SP180	0.40	0.09	0.17	0.11	0.07	0.16	0.001
E. coli OD=1 [µl]	2	CONTINLL/4	0.000	0.000	0.000	0.000	0.000	1.000	0.257
		CONTINLL/7	0.000	0.045	0.004	0.000	0.000	0.951	0.142
		CONTINLL/SP180	0.000	0.242	0.000	0.166	0.043	0.549	0.528
		CDSSTR/4	-	-	-	-	-	-	-
		CDSSTR/7	0.09	0.00	0.29	0.13	0.17	0.29	0.001
		CDSSTR/SP180	0.40	0.14	0.05	0.09	0.10	0.23	0.001
	4	CONTINLL/4	0.000	0.425	0.000	0.185	0.390	0.000	0.237
		CONTINLL/7	0.000	0.181	0.000	0.021	0.000	0.798	0.208
		CONTINLL/SP180	0.000	0.169	0.000	0.094	0.173	0.564	0.669
		CDSSTR/4	-	-	-	-	-	-	-
		CDSSTR/7	-	-	-	-	-	-	-
		CDSSTR/SP180	0.21	0.03	0.16	0.15	0.09	0.36	0.001
	8	CONTINLL/4	0.000	0.406	0.134	0.081	0.378	0.000	0.255
		CONTINLL/7	0.000	0.210	0.000	0.000	0.000	0.790	0.156
		CONTINLL/SP180	0.000	0.399	0.000	0.000	0.132	0.469	0.259

		CDSSTR/4	0.01	0.07	0.16	0.16	0.13	0.43	0.002
		CDSSTR/7	0.36	0.07	0.16	0.11	0.08	0.22	0.000
		CDSSTR/SP180	0.41	0.12	0.08	0.08	0.10	0.21	0.001
	12	CONTINLL/4	0.000	0.554	0.000	0.129	0.317	0.000	0.275
		CONTINLL/7	0.000	0.362	0.000	0.036	0.000	0.602	0.216
		CONTINLL/SP180	0.000	0.344	0.000	0.017	0.148	0.492	0.237
		CDSSTR/4	-	-	-	-	-	-	-
		CDSSTR/7	0.31	0.05	0.19	0.12	0.09	0.23	0.000
		CDSSTR/SP180	0.42	0.14	0.05	0.10	0.10	0.22	0.001

[1] normalized root mean square deviation. The NRMSD value less than 0.1 suggests a good fit of anoplin CD spectrum to the reference set, and NRSMD above 0.5 suggests that the peptide CD spectrum does not fit well to the CD spectra of the reference set. But low NRMSD do not ensure that the analysis is accurate [47].

Table S3. Fractions of secondary structures in $(KFF)_3K$ in different surroundings calculated from DichroWeb [47,48]. The experimental CD spectra are shown in Figure 4 in the main text and Figure S6.

$(KFF)_3K$		Calculated secondary structure fractions							NRMSD[1]
		Method / Reference set.	α-helix regular	α-helix distorted	β-sheet regular	β-sheet distorted	Turns	Unordered	
buffer		CONTINLL/4	0.000	0.421	0.000	0.044	0.349	0.187	0.209
		CONTINLL/7	0.000	0.364	0.000	0.009	0.000	0.627	0.105
		CONTINLL/SP180	0.000	0.221	0.000	0.051	0.140	0.589	0.496
		CDSSTR/4	-	-	-	-	-	-	-
		CDSSTR/7	0.14	-.00	0.28	0.13	0.20	0.23	0.001
		CDSSTR/SP180	0.41	0.14	0.06	0.08	0.10	0.21	0.001
SDS [mM]	1	CONTINLL/4	0.000	0.508	0.000	0.029	0.000	0.463	0.497
		CONTINLL/7	0.000	0.343	0.000	0.000	0.000	0.657	0.317
		CONTINLL/SP180	0.000	0.222	0.000	0.129	0.254	0.396	0.459
		CDSSTR/4	0.23	0.07	0.19	0.12	0.11	0.25	0.000
		CDSSTR/7	0.41	0.12	0.11	0.09	0.04	0.24	0.000
		CDSSTR/SP180	0.36	0.06	0.16	0.12	0.07	0.22	0.000
	5	CONTINLL/4	0.000	0.000	0.000	1.000	0.000	0.000	0.625
		CONTINLL/7	0.000	0.589	0.000	0.317	0.000	0.094	0.422
		CONTINLL/SP180	0.000	0.000	0.000	0.000	0.623	0.377	0.588
		CDSSTR/4	0.36	0.07	0.16	0.12	0.08	0.21	0.000
		CDSSTR/7	0.38	0.09	0.13	0.10	0.06	0.24	0.000
		CDSSTR/SP180	0.43	0.10	0.07	0.08	0.10	0.21	0.000
	10	CONTINLL/4	0.000	0.591	0.000	0.409	0.000	0.000	0.493
		CONTINLL/7	0.000	0.624	0.000	0.376	0.000	0.000	0.421
		CONTINLL/SP180	0.000	0.000	0.000	0.000	1.000	0.000	0.344
		CDSSTR/4	0.38	0.07	0.15	0.11	0.08	0.21	0.000
		CDSSTR/7	0.38	0.08	0.13	0.10	0.06	0.24	0.000
		CDSSTR/SP180	0.38	0.08	0.13	0.11	0.08	0.22	0.000
	30	CONTINLL/4	0.000	0.613	0.000	0.387	0.000	0.000	0.627
		CONTINLL/7	0.000	0.617	0.000	0.383	0.000	0.000	0.467

		CONTINLL/SP180	0.000	0.115	0.000	0.000	0.556	0.329	0.590
		CDSSTR/4	0.35	0.06	0.16	0.12	0.10	0.22	0.000
		CDSSTR/7	0.39	0.10	0.13	0.10	0.06	0.22	0.000
		CDSSTR/SP180	0.40	0.10	0.12	0.09	0.08	0.21	0.000
DPC [mM]	0.5	CONTINLL/4	0.050	0.697	0.000	0.253	0.000	0.000	0.429
		CONTINLL/7	0.162	0.619	0.000	0.219	0.000	0.000	0.154
		CONTINLL/SP180	0.000	0.349	0.000	0.000	0.214	0.437	0.404
		CDSSTR/4	0.39	0.08	0.14	0.10	0.07	0.21	0.000
		CDSSTR/7	0.40	0.10	0.10	0.08	0.07	0.24	0.000
		CDSSTR/SP180	0.41	0.10	0.10	0.09	0.10	0.22	0.000
	2	CONTINLL/4	0.000	0.078	0.922	0.000	0.000	0.000	0.350
		CONTINLL/7	0.000	0.000	1.000	0.000	0.000	0.000	0.235
		CONTINLL/SP180	0.000	0.323	0.000	0.050	0.131	0.497	0.319
		CDSSTR/4	0.37	0.07	0.17	0.11	0.07	0.21	0.000
		CDSSTR/7	0.40	0.10	0.12	0.09	0.06	0.23	0.000
		CDSSTR/SP180	0.41	0.13	0.07	0.08	0.10	0.21	0.001
	5	CONTINLL/4	0.000	0.000	1.000	0.000	0.000	0.000	0.578
		CONTINLL/7	0.000	0.589	0.411	0.000	0.000	0.000	0.672
		CONTINLL/SP180	0.000	0.508	0.000	0.000	0.306	0.186	0.342
		CDSSTR/4	0.22	0.11	0.11	0.11	0.16	0.29	0.001
		CDSSTR/7	0.38	0.17	0.11	0.09	0.06	0.16	0.001
		CDSSTR/SP180	0.43	0.11	0.08	0.08	0.10	0.21	0.001
	10	CONTINLL/4	0.000	0.000	0.000	0.000	1.000	0.000	0.556
		CONTINLL/7	0.000	0.000	1.000	0.000	0.000	0.000	0.556
		CONTINLL/SP180	0.000	0.000	0.000	0.069	0.931	0.000	0.781
		CDSSTR/4	0.25	0.02	0.28	0.14	0.12	0.20	0.001
		CDSSTR/7	0.40	0.14	0.10	0.07	0.10	0.19	0.001
		CDSSTR/SP180	0.39	0.10	0.16	0.11	0.07	0.17	0.001
POPC:POPG (3:1) [mM]	0.1	CONTINLL/4	0.000	0.000	0.000	0.000	0.000	1.000	0.241
		CONTINLL/7	0.000	0.295	0.705	0.000	0.000	0.000	0.254
		CONTINLL/SP180	0.000	0.167	0.000	0.028	0.184	0.622	0.583
		CDSSTR/4	0.36	0.07	0.17	0.11	0.08	0.22	0.000
		CDSSTR/7	0.41	0.11	0.11	0.09	0.05	0.24	0.000
		CDSSTR/SP180	0.39	0.11	0.08	0.08	0.12	0.22	0.001
	0.5	CONTINLL/4	0.000	0.000	1.000	0.000	0.000	0.000	0.768
		CONTINLL/7	0.000	0.000	0.903	0.097	0.000	0.000	0.768
		CONTINLL/SP180	0.000	0.000	0.651	0.165	0.060	0.124	0.379
		CDSSTR/4	-	-	-	-	-	-	-
		CDSSTR/7	-	-	-	-	-	-	-
		CDSSTR/SP180	-	-	-	-	-	-	-
POPC:POPE (3:1) [mM]	0.1	CONTINLL/4	0.000	0.494	0.000	0.073	0.433	0.000	0.408
		CONTINLL/7	0.345	0.590	0.000	0.065	0.000	0.000	0.312
		CONTINLL/SP180	0.000	0.116	0.000	0.052	0.170	0.662	0.542

		CDSSTR/4	0.38	0.08	0.15	0.11	0.07	0.20	0.000
		CDSSTR/7	0.40	0.12	0.11	0.09	0.07	0.21	0.001
		CDSSTR/SP180	0.43	0.13	0.06	0.07	0.11	0.18	0.001
	0.5	CONTINLL/4	0.000	0.000	0.000	1.000	0.000	0.000	0.494
		CONTINLL/7	0.503	0.497	0.000	0.000	0.000	0.000	0.447
		CONTINLL/SP180	-	-	-	-	-	-	-
		CDSSTR/4	0.49	0.12	0.07	0.06	0.05	0.22	0.001
		CDSSTR/7	0.41	0.11	0.13	0.10	0.08	0.18	0.001
		CDSSTR/SP180	0.47	0.11	0.07	0.11	0.06	0.17	0.001
LPS [µM]	10	CONTINLL/4	0.000	0.366	0.000	0.214	0.421	0.000	0.312
		CONTINLL/7	0.000	0.000	0.000	0.301	0.699	0.000	0.364
		CONTINLL/SP180	0.000	0.179	0.000	0.076	0.238	0.507	0.512
		CDSSTR/4	0.02	0.15	0.18	0.14	0.26	0.24	0.005
		CDSSTR/7	0.33	0.08	0.15	0.10	0.10	0.24	0.001
		CDSSTR/SP180	-.02	0.04	0.17	0.22	0.03	0.52	0.003
	20	CONTINLL/4	0.000	0.728	0.000	0.110	0.162	0.000	0.605
		CONTINLL/7	0.000	0.617	0.000	0.066	0.000	0.317	0.281
		CONTINLL/SP180	0.000	0.476	0.000	0.000	0.348	0.177	0.470
		CDSSTR/4	0.19	0.10	0.17	0.14	0.17	0.25	0.000
		CDSSTR/7	0.40	0.10	0.12	0.09	0.07	0.23	0.000
		CDSSTR/SP180	0.42	0.12	0.07	0.08	0.10	0.22	0.001
	50	CONTINLL/4	0.000	0.126	0.118	0.087	0.061	0.608	0.474
		CONTINLL/7	0.000	0.065	0.029	0.042	0.000	0.863	0.474
		CONTINLL/SP180	0.000	0.000	0.490	0.188	0.101	0.221	0.299
		CDSSTR/4	0.05	0.03	0.34	0.22	0.10	0.28	0.003
		CDSSTR/7	0.34	0.05	0.15	0.11	0.08	0.27	0.001
		CDSSTR/SP180	-	-	-	-	-	-	-
	100	CONTINLL/4	0.000	0.127	0.191	0.144	0.129	0.410	0.289
		CONTINLL/7	0.000	0.064	0.099	0.088	0.000	0.749	0.289
		CONTINLL/SP180	1.000	0.000	0.000	0.000	0.000	0.000	0.727
		CDSSTR/4	0.01	0.02	0.15	0.15	0.23	0.41	0.005
		CDSSTR/7	0.20	0.04	0.17	0.13	0.17	0.29	0.001
		CDSSTR/SP180	-	-	-	-	-	-	-
E. coli OD=1 [µl]	2	CONTINLL/4	0.000	0.725	0.000	0.000	0.275	0.000	0.295
		CONTINLL/7	0.000	0.000	1.000	0.000	0.000	0.000	0.222
		CONTINLL/SP180	0.000	0.398	0.000	0.000	0.209	0.393	0.301
		CDSSTR/4	0.36	0.07	0.16	0.11	0.08	0.21	0.000
		CDSSTR/7	0.38	0.12	0.13	0.10	0.06	0.21	0.000
		CDSSTR/SP180	0.39	0.10	0.09	0.10	0.10	0.22	0.000
	4	CONTINLL/4	0.000	0.402	0.000	0.147	0.452	0.000	0.417
		CONTINLL/7	0.000	0.371	0.000	0.197	0.431	0.000	0.331
		CONTINLL/SP180	0.000	0.000	0.000	0.427	0.573	0.000	0.605
		CDSSTR/4	0.35	0.07	0.18	0.12	0.08	0.20	0.001

	CDSSTR/7	0.40	0.10	0.13	0.10	0.08	0.18	0.001
	CDSSTR/SP180	0.43	0.13	0.04	0.08	0.12	0.19	0.001
8	CONTINLL/4	0.179	0.545	0.000	0.005	0.271	0.000	0.380
	CONTINLL/7	0.184	0.459	0.000	0.000	0.000	0.358	0.343
	CONTINLL/SP180	0.000	0.234	0.000	0.000	0.197	0.569	0.578
	CDSSTR/4	0.41	0.08	0.14	0.10	0.07	0.19	0.001
	CDSSTR/7	0.42	0.11	0.11	0.09	0.08	0.20	0.000
	CDSSTR/SP180	0.43	0.12	0.06	0.07	0.12	0.20	0.001
12	CONTINLL/4	0.000	0.503	0.497	0.000	0.000	0.000	0.285
	CONTINLL/7	0.000	0.439	0.561	0.000	0.000	0.000	0.200
	CONTINLL/SP180	0.000	0.276	0.000	0.054	0.133	0.537	0.361
	CDSSTR/4	-	-	-	-	-	-	-
	CDSSTR/7	0.33	0.05	0.18	0.11	0.09	0.24	0.000
	CDSSTR/SP180	0.39	0.10	0.10	0.09	0.10	0.22	0.000

[1] normalized root mean square deviation. The NRMSD value less than 0.1 suggests a good fit of anoplin CD spectrum to the reference set, and NRSMD above 0.5 suggests that the peptide CD spectrum does not fit well to the CD spectra of the reference set. But low NRMSD do not ensure that the analysis is accurate [47].
